# Geographical Variation in Antimalarial Drug Resistance Marker Prevalence across the Southern African Elimination Eight Region

**DOI:** 10.4269/ajtmh.26-0299

**Published:** 2026-10-06

**Authors:** Jaishree Raman, Andrés Aranda-Díaz, Maxwell Mabona, Mukosha Chisenga, Maria Florinda João, Domingos Jandondo, Pedro Rafael Dimbu, Nomcebo Nhlengethwa, Sabelo V. Dlamini, Lydia Eloff, Stark Katokele, Davis R. Mumbengegwi, Qedusizi Nyawo, Mbavhalelo Shandukani, Sydney Mwanza, Moonga Hawela, Simone Boene, Arlindo Chidimatembue, Bernardete Rafael, Eduard Rovira-Vallbona, Brighton Mangena, Sonja B. Lauterbach, Takalani I. Makhanthisa, Hazel Gwarinda, Blaženka D. Letinić, Faith De Amaral, Isobel Routledge, Beatriz Arregui-Gallego, Mishalan Moodley, Jonathan Featherston, Power B. Tshikae, Arshad Ismail, Jose Franco Martins, Quinton Dlamini, Baltazar Candrinho, Petrina Uusiku, Ednah Baloyi, Busiku Hamainza, Alfredo Mayor, Bryan Greenhouse, Amy Wesolowski, Chadwick Sikaala, John Chimumbwa, Jennifer L. Smith

**Affiliations:** ^1^ Laboratory for Antimalarial Resistance Monitoring and Malaria Operational Research, Centre of Emerging Zoonotic and Parasitic Diseases, National Institute for Communicable Diseases, Johannesburg, South Africa;; ^2^ Wits Research Institute for Malaria, University of Witwatersrand, Johannesburg, South Africa;; ^3^ University of Pretoria Institute for Sustainable Malaria Control, University of Pretoria, Pretoria, South Africa;; ^4^ EPPIcenter Research Program, Department of Medicine, University of California, San Francisco, California, USA;; ^5^ Malaria Elimination Initiative, Global Health Group, University of California, San Francisco, California, USA;; ^6^ ISGlobal, Barcelona, Spain;; ^7^ SADC Malaria Elimination Eight Secretariat, Windhoek, Namibia;; ^8^ Programa Nacional do Controlo da Malária, Luanda, Angola;; ^9^ Instituto Nacional de Investigação em Saúde, Luanda, Angola;; ^10^ National Malaria Programme, Manzini, Eswatini;; ^11^ Department of Environmental Health Sciences, University of Eswatini, Mbabane, Eswatini;; ^12^ Department of Biochemistry, Microbiology, and Biotechnology, School of Science, University of Namibia, Windhoek, Namibia;; ^13^ Malaria Operational Research Program, Multidisciplinary Research Services, University of Namibia, Windhoek, Namibia;; ^14^ Ministry of Health and Social Services, National Vector-Borne Disease Control Program, Windhoek, Namibia;; ^15^ KwaZulu-Natal Provincial Malaria Control Program, Jozini, South Africa;; ^16^ National Department of Health, Pretoria, South Africa;; ^17^ National Health Research and Training Institute, Ndola, Zambia;; ^18^ National Malaria Elimination Centre, Ministry of Health, Lusaka, Zambia;; ^19^ Centro de Investigação em Saúde de Manhiça, Manhiça, Mozambique;; ^20^ Facultat de Medicina i Ciències de la Salut, Universitat de Barcelona, Barcelona, Spain;; ^21^ Programa Nacional do Controlo da Malária, Maputo, Mozambique;; ^22^ Sequencing Core Facility, National Institute for Communicable Diseases, Johannesburg, South Africa;; ^23^ Faculdade de Medicina, Universidade Eduardo Mondlane, Maputo, Mozambique;; ^24^ Spanish Consortium for Research in Epidemiology and Public Health, Madrid, Spain;; ^25^ Department of Epidemiology, Johns Hopkins University Bloomberg School of Public Health, Baltimore, Maryland, USA;; ^26^ Department of Epidemiology & Biostatistics, University of California, San Francisco, California, USA

## Abstract

Global efforts to control and eliminate malaria are threatened by antimalarial drug resistance. The WHO recommends molecular surveillance of resistance markers as a complementary approach to therapeutic efficacy studies. Here, the first regional analysis of antimalarial resistance markers across six southern African countries spanning diverse transmission intensities and geographies is reported. Dried blood spots, collected from rapid diagnostic test-positive individuals in Angola, Eswatini, Namibia, and Zambia in 2023, Mozambique in 2022, and South Africa between 2022 and 2024 using a standardized collection method, were analyzed using a *Plasmodium falciparum*-targeted amplicon sequencing protocol. The distribution of resistance markers was spatially heterogeneous. Validated markers of artemisinin partial resistance (ART-R) were rare. However, more than 30% of samples from eastern Namibia and Zambia’s Western and Central Provinces carried the candidate *kelch13* P441L ART-R marker. Other ART-R markers (*kelch13* R515K, P553L, P574L, A675V) were detected at a prevalence rate <1.3% in all countries except Mozambique, where these markers were absent. The wild-type multidrug resistance 1 N86 allele, potentially associated with reduced lumefantrine susceptibility, was near fixation (>99%) across all countries, whereas the chloroquine resistance marker K76T, in the chloroquine resistance transporter (*crt*) was nearly absent in most districts. The sulfadoxine–pyrimethamine resistance dihydropteroate synthase (*dhps*)-dihydrofolate reductase quintuple mutation was approaching fixation (>95%) in 35% of districts. Northern Angola was a clear outlier, with a lower *dhps* K540E prevalence (≤20%) but a higher *crt* K76T prevalence (48% to 56%). This pronounced spatial heterogeneity underscores the need for timely high-resolution local resistance data generation and sharing to safeguard antimalarial drug efficacy and guide malaria control and elimination strategies across the region.

## INTRODUCTION

The already diminishing progress against malaria since 2016 was further exacerbated by the coronavirus disease 2019 pandemic.^1^^,^^2^ There were ∼9 million more cases in 2024 compared with 2023, the majority of which were from sub-Saharan Africa.^3^ Reasons for this stagnation and, in some cases, reversal in progress include reduced funding for malaria control, disruptions in the delivery of routine malaria case management and vector control services, the spread of invasive vector species, and reduced efficacy of core interventions as a consequence of vector behavior changes or genetic evolution of malaria parasites and vectors.^2^^,^^4^

Antimalarial drug-resistant *Plasmodium falciparum* has historically undermined malaria control efforts in sub-Saharan Africa. During the expansion of chloroquine resistance in the region, some countries experienced a 2- to 3-fold increase in malaria deaths and hospital admissions.^5^ Similarly, the establishment of sulfadoxine–pyrimethamine (SP)-resistant parasites in southern Africa played a pivotal role in the 1999/2000 malaria outbreak, which resulted in South Africa reporting more than 60,000 cases.^6^ Retrospective genomic analyses of African chloroquine- and SP-resistant parasites revealed a close genetic relationship between parasites that emerged along the Thai–Cambodia border and those present in Africa, confirming the spread of these parasites from Southeast Asia through selective genetic sweeps.^7^^–^^9^ The widespread replacement of these failing antimalarials with highly effective artemisinin-based combination therapies (ACTs) played a central role in the significant reductions in malaria burden observed between 2000 and 2015.^10^ However, the recent de novo emergence of artemisinin partial resistance (ART-R) in multiple African countries threatens the sustained efficacy of ACTs and the achievement of global targets outlined in the Global Strategy for Malaria.^4^^,^^11^ Widespread resistance to ACTs in Africa is estimated to result in millions of additional malaria cases, thousands of excess deaths, and annual economic losses ranging from $100 million to $1.1 billion.^12^ Urgent action is therefore needed to protect the sustained efficacy of ACTs while new non-artemisinin-based antimalarials are brought to market.^13^ Although the WHO recommends the routine monitoring of validated molecular markers of resistance, molecular data from southern Africa remain scarce.^14^^,^^15^ A major contributing factor is limited regional capacity to generate, analyze, and translate molecular data into actionable information for effective programmatic decision-making.

The Southern African Development Community’s Malaria Elimination Eight (SADC E8), a consortium of eight southern African countries (Angola, Botswana, Eswatini, Mozambique, Namibia, South Africa, Zambia, and Zimbabwe), was established to promote collaborative efforts toward malaria elimination.^16^ Strengthening malaria molecular surveillance capacity and generating relevant, timely molecular data in the SADC E8 is critical to enhancing preparedness and identifying emerging threats to ongoing control and elimination efforts. Therefore, five SADC E8 countries (Angola, Eswatini, Namibia, South Africa, and Zambia) together with the SADC E8 Secretariat and technical partners, including the University of California San Francisco (UCSF), Johns Hopkins University, and the South African National Institute for Communicable Diseases (NICD), implemented a regional malaria genomic epidemiology initiative, the Genomics for Malaria in the SADC E8 (GenE8). This initiative was designed to generate programmatically relevant genomic data across the five participating countries, promote prompt data sharing, and encourage the incorporation of genomic surveillance into routine malaria surveillance activities.

The Regional GenE8 (RegGenE8) study, embedded within the GenE8 initiative, was focused on generating malaria genomic data for the two high-burden countries (Angola and Zambia) and three low-burden countries (Eswatini, Namibia, and South Africa), using harmonized sampling frameworks and centralized sequencing. Concurrently, through the GenMoz project, Mozambique generated malaria molecular data comparable to that produced through the RegGenE8 study.^17^ By integrating and reanalyzing data from these two projects, we report the first regional synthesis of antimalarial drug resistance marker presence and distribution across six southern African countries, highlighting their implications for malaria control and elimination efforts.

## MATERIALS AND METHODS

### Study settings.

The RegGenE8 study was conducted across five malaria-endemic southern African countries, Angola, Eswatini, Namibia, South Africa, and Zambia, whereas the GenMoz study was conducted across nine provinces in Mozambique (Figure 1); detailed descriptions of each country setting are provided in the respective country-level analyses.^20^^–^^25^ Malaria transmission intensity in these six countries is heterogeneous, with a general north-to-south decrease in transmission intensity. Although transmission occurs year-round, peak transmission typically coincides with the hot, rainy season from December to May, and seasonality becomes more pronounced farther south from the equator. *Plasmodium falciparum* is the dominant malaria parasite across the region, with *Anopheles funestus*, *Anopheles gambiae* s.s., and *Anopheles arabiensis* serving as the predominant vectors. Indoor residual spraying and long-lasting insecticidal nets are the primary vector control interventions.

**Figure 1.**
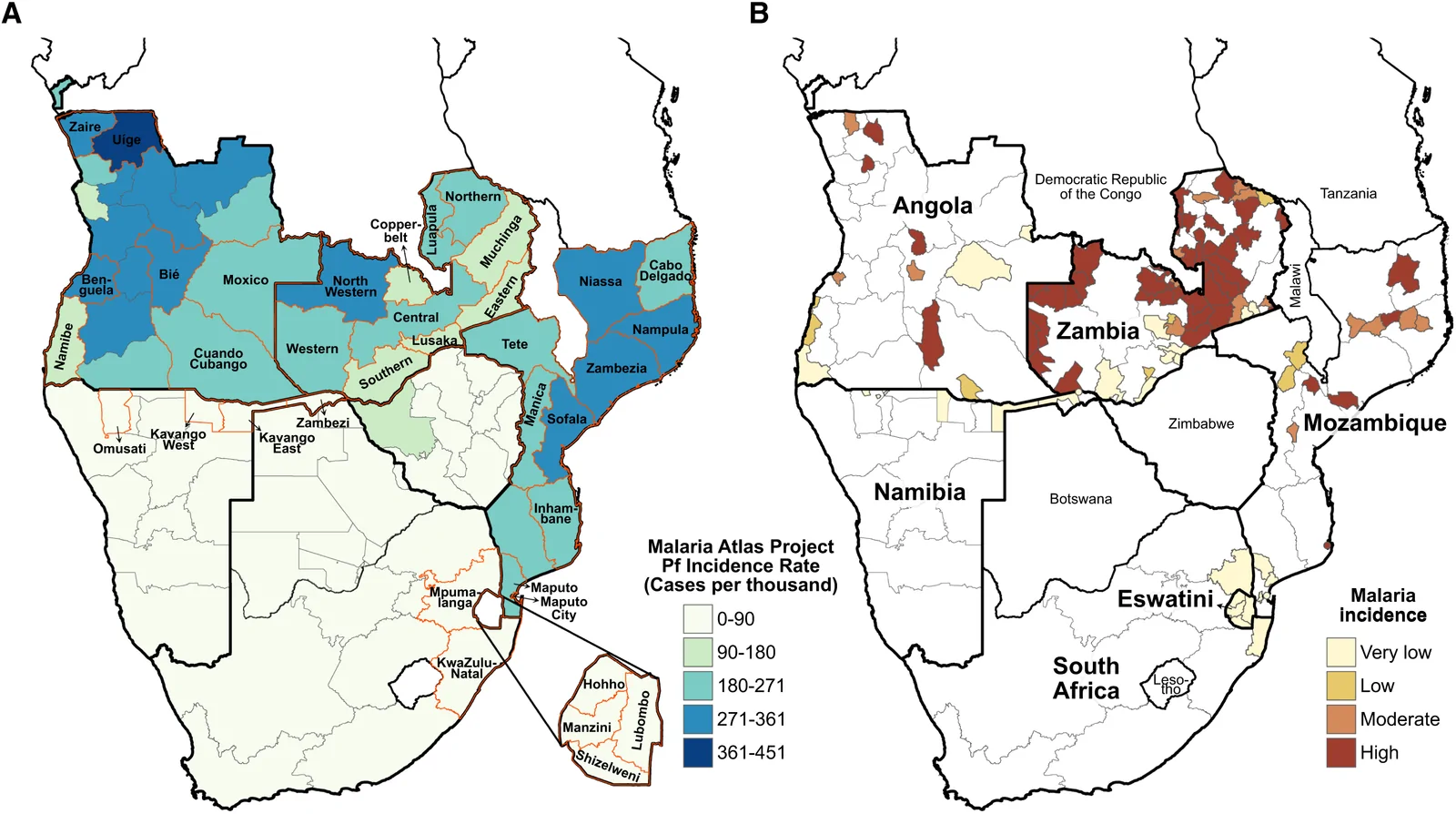
(**A**) Map of the Southern Africa region revealing the provinces or regions from which samples were collected, highlighted in orange. All provinces in the Elimination Eight countries are colored by malaria incidence rate on the basis of 2023 Malaria Atlas Project data.^18^ (**B**) Districts from the participating Elimination Eight countries included in this manuscript, stratified by malaria incidence according to the WHO framework for malaria elimination on the basis of annual parasite incidence per 1,000 population: very low (<100), low (100–249), moderate (250–449), and high (≥450).^19^ Incidence data are country-reported for 2023, except Mozambique (2022) and South Africa (2022–2024).

Rapid diagnostic tests (RDTs) and microscopy are the primary diagnostic tools used in the participating countries. Mozambique, South Africa, and Zambia use histidine-rich protein 2 (HRP2)-based *falciparum*-specific RDTs, whereas in Eswatini and Namibia, RDTs that detect *P*. *falciparum* HRP2 and pan-*Plasmodium* lactate dehydrogenase (LDH) are recommended. Angola uses a *P*. *falciparum* HRP2 and *Plasmodium vivax* LDH-based RDT. Artemether–lumefantrine is a first-line ACT for uncomplicated malaria throughout the region, with artesunate–amodiaquine serving as an alternate first-line treatment option in Angola and Mozambique. Dihydroartemisinin–piperaquine (DHAP) is also listed as a first-line treatment option in Angola. Severe malaria cases are treated with intravenous artesunate in all six countries, with intravenous quinine reserved as a second-line option in certain countries. The transmission-blocking antimalarial, single low-dose primaquine, is part of the standard treatment of uncomplicated malaria in elimination districts of Eswatini, Namibia, South Africa, and more recently, in selected very low-transmission districts in Zambia.^26^ Intermittent preventive treatment (IPT) in pregnancy with SP is standard practice in Angola, Mozambique, and Zambia, whereas seasonal malaria control with SP–amodiaquine has been implemented in Nampula Province in Mozambique.^20^^,^^24^^,^^25^^,^^27^ Mass drug administration (MDA) with DHAP has been evaluated in community-randomized trials in Southern Province, Zambia (2014–2016), and is now a routine activity in elimination districts. Reactive focal mass administration of DHAP was trialed in the Zambezi Region of Namibia (2017) and in Eswatini (2015–2017) but has not been adopted as national policy in either country.^28^^–^^30^ Additional details on country-specific study settings are described in previously published country-level analyses.^20^^–^^25^

### Study design, sampling, and data collection.

The RegGenE8 study was a cross-sectional, multisite healthcare facility-based observational study embedded within the national malaria surveillance systems of the six participating countries. The study was conducted in Angola (seven of 18 provinces), Eswatini (all regions), Namibia (all endemic regions), and Zambia (all 10 provinces) in 2023 and in South Africa (two of three endemic provinces) from 2022 to 2024. In Angola, logistical constraints limited sampling to a stratified subset of endemic provinces, whereas in South Africa, the endemic province of Limpopo did not participate. Healthcare facilities were selected to capture the range of transmission settings and geographic regions within each country. In the GenMoz study, healthcare facility-based surveys were conducted in 10 of the 11 Mozambican provinces during the 2021 and 2022 rainy seasons and in two low-transmission provinces during the dry season. This report includes only GenMoz survey data from 2022, when participant inclusion criteria varied by province. In Maputo Province, individuals >6 months of age provided samples; in Inhambane, Zambézia, and Sofala Provinces, participation was restricted to children aged between 2 and 10 years; in areas undergoing seasonal malaria chemoprevention in Nampula Province, only children between 3 months and 5 years of age were recruited; and samples from Tete and Cabo Delgado Provinces were collected during the 2022 therapeutic efficacy studies (TES), where only children between 6 months and 5 years of age with a parasite density >2,500/*µ*L by microscopy were included.^25^

Sampling strategies and data collection methodologies are described in detail in the country-specific publications, but a summary is provided here for completeness.^20^^–^^25^ Across all study sites, symptomatic *P. falciparum* cases confirmed by RDT or microscopy were recruited. Cases identified through passive case detection in all participating countries, as well as reactive or proactive screening in South Africa, Eswatini, and southern Zambia, were included in the study. More than 98% of the samples collected during reactive or proactive screening in South Africa were from asymptomatic individuals. Symptoms were not recorded in Eswatini or Zambia during active case detection activities. Finger-prick dried blood spots (DBS) or used RDTs were collected from consenting individuals (see Disclosures), labeled with unique barcodes linking survey and laboratory data, air-dried, and stored individually with desiccant in sealed bags until shipment for sequencing. RegGenE8 samples were shipped to either the NICD or UCSF, whereas GenMoz samples were transported to the Manhiça Health Research Center, Manhiça, Mozambique, or to ISGlobal in Barcelona, Spain. Demographic, clinical, and travel-history data were collected using electronic or paper-based questionnaires. Written informed consent was obtained from all participants, except in South Africa (see Disclosures). Patients with severe malaria, severe malnutrition, or febrile illness attributable to nonmalarial diseases were excluded from the studies.

Country-specific sampling strategies and numbers of sequenced samples are summarized in Table 1. These harmonized datasets were used for downstream analyses, including mapping the prevalence and distribution of antimalarial resistance markers. Sample numbers reported here may differ slightly from those in previously published country-specific analyses because all available RegGen8 and GenMoz sequences from the six participating countries were reanalyzed for the present study using standardized quality filters and cutoffs.

**Table 1:** Sociodemographic characteristics of study participants in the six participating Elimination Eight countries

Variable	Overall	Angola	Eswatini	Mozambique	Namibia	South Africa	Zambia
Reference	–	João et al. 2026^20^	Nhlengethwa et al. 2026^21^	Boene et al. 2025^25^	Eloff et al. 2026^22^	Raman et al. 2026^23^	Aranda-Díaz et al. 2026^24^
Sampling years	–	2023	2023	2022	2023	2022–2024	2023
Health facilities	–	14 (7 provinces)	54 (4 regions)	66 (10 provinces)	16 (5 regions)	127 (2 provinces)	61 (10 provinces)
Total samples	6,485	781	425	864	236	1,774	2,405
Characteristic							
Sex*							
Female	2,743 (46%)	413 (53%)	94 (22%)	423 (50%)	104 (44%)	509 (41%)	1,200 (50%)
Male	3,192 (54%)	368 (47%)	331 (78%)	430 (50%)	132 (56%)	726 (59%)	1,205 (50%)
Unknown	550	0	0	11	0	539	0
Age group (years)*							
0–4	1,102 (17%)	176 (23%)	12 (2.9%)	481 (56%)	20 (8.5%)	163 (9.3%)	250 (10%)
5–14	1,497 (23%)	167 (21%)	140 (33%)	20 (2.3%)	52 (22%)	427 (24%)	691 (29%)
15–24	1,256 (19%)	78 (10.0%)	138 (33%)	30 (3.5%)	47 (20%)	614 (35%)	349 (15%)
25–39	606 (9.4%)	44 (5.6%)	52 (12%)	16 (1.9%)	26 (11%)	322 (18%)	146 (6.1%)
40–59	1,841 (29%)	307 (39%)	58 (14%)	301 (35%)	81 (34%)	156 (8.9%)	938 (39%)
60+	140 (2.2%)	9 (1.2%)	20 (4.8%)	7 (0.8%)	10 (4.2%)	66 (3.8%)	28 (1.2%)
Unknown	43	0	5	9	0	26	3
Occupation*							
Agriculture	736 (20%)	75 (10%)	198 (47%)	NA	12 (5.2%)	21 (12%)	430 (20%)
Minor	1,291 (35%)	266 (36%)	18 (4.3%)	NA	35 (15%)	14 (8.1%)	958 (44%)
Other	303 (8.1%)	46 (6.2%)	41 (9.7%)	NA	13 (5.6%)	63 (37%)	140 (6.5%)
Student	799 (21%)	317 (43%)	90 (21%)	NA	92 (40%)	7 (4.1%)	293 (14%)
Unemployed	604 (16%)	36 (4.9%)	76 (18%)	NA	79 (34%)	67 (39%)	346 (16%)
Unknown	2,752	41	2	864	5	1,602	238
Collection method*							
Passive	5,502 (89%)	781 (100%)	398 (94%)	864 (100%)	236 (100%)	1,112 (65%)	2,111 (96%)
Active/reactive	709 (11%)	0 (0%)	27 (6.4%)	0 (0%)	0 (0%)	590 (35%)	92 (4.2%)
Unknown	274	0	0	0	0	72	202
Travel history*							
No travel	3,637 (69%)	725 (93%)	277 (65%)	NA	215 (92%)	167 (11%)	2,253 (98%)
Domestic	148 (2.8%)	55 (7.0%)	32 (7.5%)	NA	10 (4.3%)	11 (0.7%)	40 (1.7%)
International	1,346 (27%)	1 (0.1%)	119 (27%)	NA	9 (3.7%)	1,336 (88%)	2 (<0.1%)
Unknown	1,239	0	0	864	2	263	110

* Characteristic differed significantly between countries (χ^2^ test; *P* <0.001).

### Sample processing and genomic assays.

Dried blood spot and RDT samples were processed and sequenced using previously described harmonized protocols.^20^^–^^25^^,^^31^ Genomic DNA was extracted using the Chelex-Tween 20 protocol, with parasitemia confirmed and quantified using a quantitative polymerase chain reaction (qPCR) assay targeting the *P*. *falciparum* var gene acidic terminal sequence in the RegGenE8 study or the 18S ribosomal RNA subunit gene in the GenMoz study.^32^^–^^34^ The extracted, quantified DNA was stored at –20°C until sequencing. Amplicon-based sequencing was performed using the MAD^4^HatTeR (Multiplex Amplicons for Drug, Diagnostic, Diversity, and Differentiation Haplotypes using Targeted Resequencing) multiplex panel and protocol, an adaptation of the CleanPlex® Amplicon Sequencing Technology (Paragon Genomics, Inc., Hayward, CA, USA), using the MAD^4^HatTeR primer pools D1.1, R1.1 or R1.2, and R2.1.^31^ Sequencing was conducted on Illumina MiSeq, NextSeq, or NovaSeq instruments (Illumina, Inc., San Diego, CA, USA) using 150 paired-end reads.

Allele calls were obtained using the bioinformatic pipeline specifications previously described.^22^ For GenMoz data, raw FASTQ files were downloaded from the Sequence Read Archive (National Institutes of Health, Bethesda, MD, USA) and re-run using the same pipeline version used for the RegGenE8 data. Sequences resulting from loci targeted by primer pools R1.1, R1.2, and R2.1 in the *kelch13* (PF3D7_1343700), chloroquine resistance transporter (*crt*, PF3D7_0709000), multidrug resistance 1 (*mdr1*, PF3D7_0523000), dihydropteroate synthase (*dhps*, PF3D7_0810800), dihydrofolate reductase (*dhfr*, PF3D7_0417200), coronin (PF3D7_1251200), exonuclease (*exo*, PF3D7_1362500), multidrug resistance 2 (*mdr2*, PF3D7_1447900), ferredoxin (*fd*, PF3D7_1318100), apicoplast ribosomal protein S10 (*arps10*, PF3D7_1460900), phosphoinositide-binding protein (*px1*, PF3D7_0720700), and nuclear LIM interactor-interacting factor-like phosphatase (*pph*, PF3D7_1012700) genes are reported. Allele calls output by the bioinformatic pipeline are available in a public repository in a Portable Microhaplotype Object (PMO) format (doi: 10.5281/zenodo.21438965). Portable Microhaplotype Object is a JSON-based standardized format for efficiently and losslessly storing phased targeted sequencing data. Information on the PMO project, including the ontology specification, software tools, and tutorials, is available at https://plasmogenepi.github.io/PMO_Docs/.^35^ Additional data cleaning, analysis, and visualization were conducted in R (version 4.3.2; R Foundation, Vienna, Austria). Samples with less than 10,000 reads, targets with <50 reads, and single-nucleotide polymorphisms (SNPs) with within-sample allele frequencies (WSAF)s <0.01 or ≤10 reads were excluded from the analysis. Subsequently, SNPs observed in <0.05% of samples with WSAFs <1 and SNPs observed in multiple samples but exclusively in a single sequencing run were also excluded. Library preparation batches with contaminated negative or positive controls were repeated or excluded from all analyses. Samples were considered successfully sequenced if a genotype was obtained for all 59 SNPs evaluated in the *kelch13* gene.

Multilocus haplotypes for polymorphisms in the *dhps* and *dhfr* genes (*dhps*: 436, 437, 540, 581, and 613; *dhfr*: 51, 59, 108, and 164) covered by five independent amplicon targets were identified for each sample by combining all individual microhaplotypes. Haplotypes were identified for samples with either unmixed genotypes at all targets or a single target with a mixed genotype; in the latter case, the sample was assumed to carry both haplotypes to account for the observed mixture. Samples with mixed genotypes in more than two amplicons were considered indeterminate.

To identify potential issues with sample collection and library preparation, sample similarity was estimated using the root mean square error of WSAF for highly diverse targets (pool D1.1), followed by clustering using Density-Based Spatial Clustering of Applications with Noise with ε = 0.2. Clusters of samples (at least two samples) with clonality >2 and nearly identical WSAFs across all loci, indicating that multiple DBSs were prepared using blood from a single patient, were excluded from the analysis. Multiple such clusters within a RegGenE8 health facility led to the exclusion of all data from that facility.

## STATISTICAL ANALYSES

Descriptive statistics for sex, age group (<5, 5–14, 15–24, 25–39, 40–59, and >59 years old), overnight travel in the past 2 months, and country of residence were calculated. Individual participant age was rounded down for age-group classification. To enable statistical analysis, some variables were reclassified: farming, herding, and fishing occupations were combined into a single occupational category (agricultural workers); individuals aged 25 years or older were grouped into a single category; and travel reports (within the 2 months before sample collection) were categorized as either international or domestic. Differences in sociodemographic characteristics and resistance marker prevalence between countries, as well as between successfully sequenced and qPCR-positive samples, were assessed using χ^2^ tests.

The proportion of samples carrying a mutation or haplotype was calculated as the number of samples with mixed and pure mutant genotypes divided by the total number of genotyped infections. Province- and district-level aggregate data are available in a public repository (doi: 10.5281/zenodo.21331900).

## RESULTS

### Sociodemographic characteristics of the study population.

A total of 6,485 samples were used in the present regional analysis. In higher-transmission countries (Angola, Mozambique, and Zambia), the largest proportion of samples (40%) was collected from participants aged 14 years or younger. In Eswatini and South Africa, both low-transmission countries targeting malaria elimination, most study participants were aged 15 to 39 years (Table 1). Namibia fell between these two groupings, with most study participants aged 5 to 39 years (Table 1). Sex distribution was balanced in the sample sets from Angola, Mozambique, and Zambia, whereas males predominated in the datasets from the very low-transmission countries Eswatini, Namibia, and South Africa (Table 1). Occupational information was rarely recorded in South Africa and was not collected in Mozambique; thus, these countries were excluded from the occupation analysis. Although there was a significant difference in occupations among the four remaining countries (χ^2^ test; *P* <0.001), 56% of the participants were either minors or students, with most of the participants working in the agricultural sector (Table 1). International travelers accounted for almost 90% of cases in South Africa and more than one-quarter of cases in Eswatini. In contrast, international travel was uncommon among participants with successfully sequenced samples from Angola, Namibia, and Zambia. Most samples were collected through passive case detection, with 11% collected through active and reactive case detection activities in Eswatini, South Africa, and Zambia.

### Molecular analysis.

A total of 114 individual SNPs across 12 genes were evaluated. Amplicons had a median depth of 2,303 reads (interquartile range: 750–6,965); individual per-locus depth by country is shown in Supplemental Table 1.

### Mutations in the *kelch13* gene.

A total of 15 nonsynonymous mutations were detected in the *kelch13* gene, with prevalence and distribution varying within and between countries. The *kelch13* gene was most diverse in Zambia, where 11 unique mutations were identified. Namibia, despite having the smallest sample size, had nine nonsynonymous mutations and the highest proportion of samples carrying a *kelch13* mutation. In contrast, the gene was most conserved in Eswatini, with only a single *kelch13* mutation detected over the study period (Table 2; Figure 2; Supplemental Table 2; Supplemental Figure 1).

**Table 2:** Proportion of samples with nonsynonymous mutations in markers of antimalarial drug resistance across participating Elimination Eight countries

Antimalarial Drug Resistance Marker	Angola	Eswatini	Mozambique	Namibia	South Africa	Zambia
Validated ART-R markers						
* kelch13* P553L	0% (*N* = 754)	0.97% (*N* = 412)	0% (*N* = 840)	0% (*N* = 233)	0.07% (*N* = 1,522)	0% (*N* = 2,254)
* kelch13* P574L	0.13% (*N* = 781)	0% (*N* = 424)	0% (*N* = 854)	1.27% (*N* = 236)	0.23% (*N* = 1,770)	0.67% (*N* = 2,400)
* kelch13* A675V	0% (*N* = 711)	0% (*N* = 382)	0% (*N* = 847)	1.3% (*N* = 231)	0% (*N* = 1,334)	0.1% (*N* = 2,091)
Candidate ART-R markers						
* kelch13* R515K	0% (*N* = 762)	0% (*N* = 418)	0% (*N* = 798)	0.9% (*N* = 221)	0% (*N* = 1,589)	0% (*N* = 2,214)
* kelch13* P441L	0.4% (*N* = 742)	0% (*N* = 412)	0% (*N* = 846)	32.9% (*N* = 231)	0% (*N* = 1,509)	3% (*N* = 2,235)
Marker potentially associated with reduced lumefantrine susceptibility						
* mdr1* N86	99.2% (*N* = 628)	100% (*N* = 336)	99.88% (*N* = 836)	100% (*N* = 228)	99.92% (*N* = 1,207)	100% (*N* = 1,949)
SP resistance markers						
* dhps* ISGEAA, *dhfr* IRNI (quintuple)	25.9% (*N* = 525)	93.25% (*N* = 400)	91.81% (*N* = 659)	72.02% (*N* = 218)	97.32% (*N* = 1,382)	86.21% (*N* = 1,827)
* dhps* ISGEGA, *dhfr* IRNI (sextuple)	1.71% (*N* = 525)	6% (*N* = 400)	0.61% (*N* = 659)	11.01% (*N* = 218)	0.29% (*N* = 1,382)	3.39% (*N* = 1,827)
Chloroquine resistance marker						
* crt* 72–76 CVIET	18.82% (*N* = 781)	0.47% (*N* = 425)	0.12% (*N* = 862)	0.85% (*N* = 235)	0.06% (*N* = 1,739)	0.21% (*N* = 2,405)

ART-R = artemisinin partial resistance; *crt* = chloroquine resistance transporter; *dhfr* = dihydrofolate reductase; *dhps* = dihydropteroate synthase; *mdr1* = multidrug resistance 1; SP = sulfadoxine–pyrimethamine. Mutations are shown for ART-R (*kelch13*), potential lumefantrine reduced susceptibility (*mdr1*), SP resistance (*dhfr*, *dhps*), and chloroquine resistance (*crt*, *mdr1*). Artemisinin partial resistance markers were categorized according to the current WHO classification framework as validated, candidate, or unvalidated markers of ART-R.^37^ Proportions are calculated as the number of samples with a genotype (pure or mixed) divided by the total number of genotyped infections.

**Figure 2.**
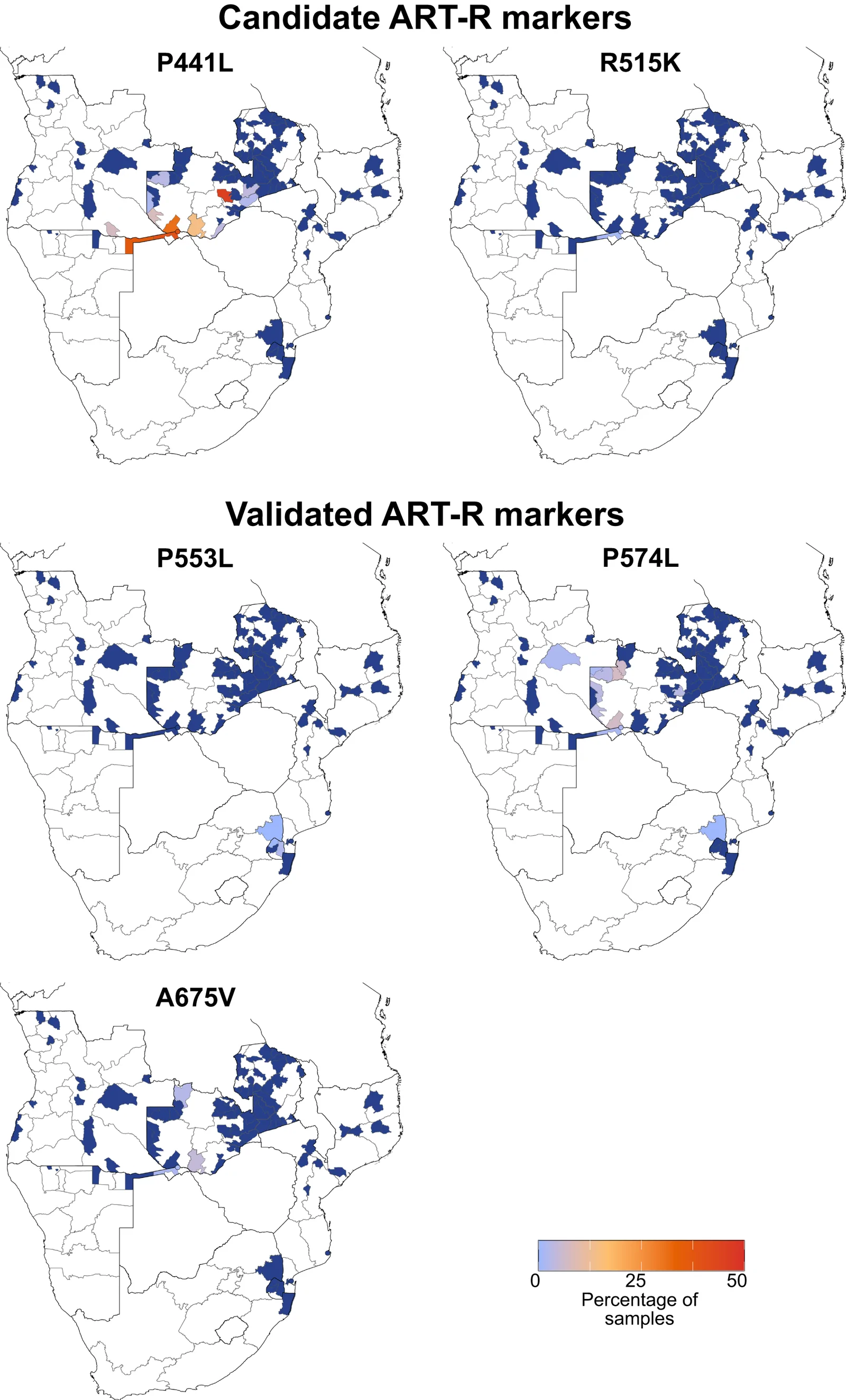
Proportion of samples with validated or candidate artemisinin partial resistance markers in *kelch13* across districts in the Elimination Eight countries participating in the Regional Genomics for Malaria in the Southern African Development Community’s Malaria Elimination Eight and GenMoz studies. Proportions are calculated as the number of samples with a genotype (pure or mixed) divided by the total number of genotyped infections. Dark blue shading indicates the absence of a marker.

The *kelch13* A578S mutation, not known to be associated with ART-R, was detected across multiple sites and transmission strata in all study countries except Eswatini but did not exceed 2.5% prevalence in any country.^36^ The validated ART-R *kelch13* P574L marker was observed in all countries except Eswatini and Mozambique. This mutation was most prevalent in three high-incidence districts in Zambia’s Western and North-Western Provinces (6.4% to 7.1%). It was also detected in a moderate-incidence district in Zambia’s Central Province, as well as in very low-incidence districts in Namibia’s Zambezi Region and Angola’s Moxico Province, both bordering Zambia’s Western or North-Western Provinces, and in South Africa’s Mpumalanga Province. The candidate ART-R marker, *kelch13* P441L, detected only in Angola, Namibia, and Zambia, reached proportions exceeding 30% in very low-incidence settings in Namibia’s Zambezi and Kavango East Regions and Zambia’s Chibombo District (Central Province), as well as in the high-incidence Sesheke District in Zambia’s Western Province. Outside these districts, *kelch13* P441L was observed at proportions less than 15% across settings with transmission ranging from very low to high, primarily along the shared border of Angola, Namibia, and Zambia. The mutation was also detected in Zambia’s Lusaka Province (Figure 2).

The uncharacterized *kelch13* P667A mutation had a similar geographical distribution to P441L, reaching proportions greater than 35% in Zambia’s high-incidence Chavuma and Zambezi Districts in North-Western Province (Figure 2). Other mutations, including *kelch13* R622T, P667S, and the validated ART-R marker A675V, were detected only in Zambia and Namibia. The *kelch13* R622T mutation exceeded 10% in districts with both very low and high incidence in Zambia’s Southern and North-Western Provinces. The *kelch13* A675V mutation was detected at low proportions (<5%) in very low-incidence districts in Zambia’s Southern Province and Namibia’s Zambezi Region, as well as in a high-incidence district in Zambia’s North-Western Province.

The validated ART-R marker *kelch13* P553L was observed in a small cluster of genetically related infections from very low-incidence settings in Eswatini, including a sample from a traveler from Mozambique and a single South African sample collected in 2023. This mutation was not observed in Mozambican samples collected in 2022. The candidate ART-R *kelch13* R515K mutation was detected only in Namibia, in less than 1% of samples. Other nonsynonymous *kelch13* mutations, including K479I, S485N, V494I, N537S, and Q613E, were observed at low proportions across multiple countries and transmission settings.

### Mutations in the *dhps* and *dhfr* genes.

The proportion of samples carrying the *dhfr* triple mutation (*dhfr* N51I, *dhfr* C59R, and *dhfr* S108N), which is strongly associated with pyrimethamine resistance, exceeded 85% in all participating countries and was close to fixation in Eswatini, South Africa, and Mozambique (Figure 3; Supplemental Figure 2; Supplemental Table 3). However, in districts in southwestern Angola and western Namibia, the *dhfr* N51I/C59/S108N haplotype was detected at much lower frequencies, present in just over 20% of samples. In Zambia’s Pemba District, Southern Province, three *dhfr* haplotypes (N51I/C59R/S108N, N51I/C59/S108N, and N51/C59R/S108N) were co-circulating at substantial frequencies. The *dhfr* I164L mutation, associated with an ∼1,000% increase in pyrimethamine resistance, remains extremely rare in the region and was detected in only two samples from Muchinga Province in Zambia.^38^

**Figure 3.**
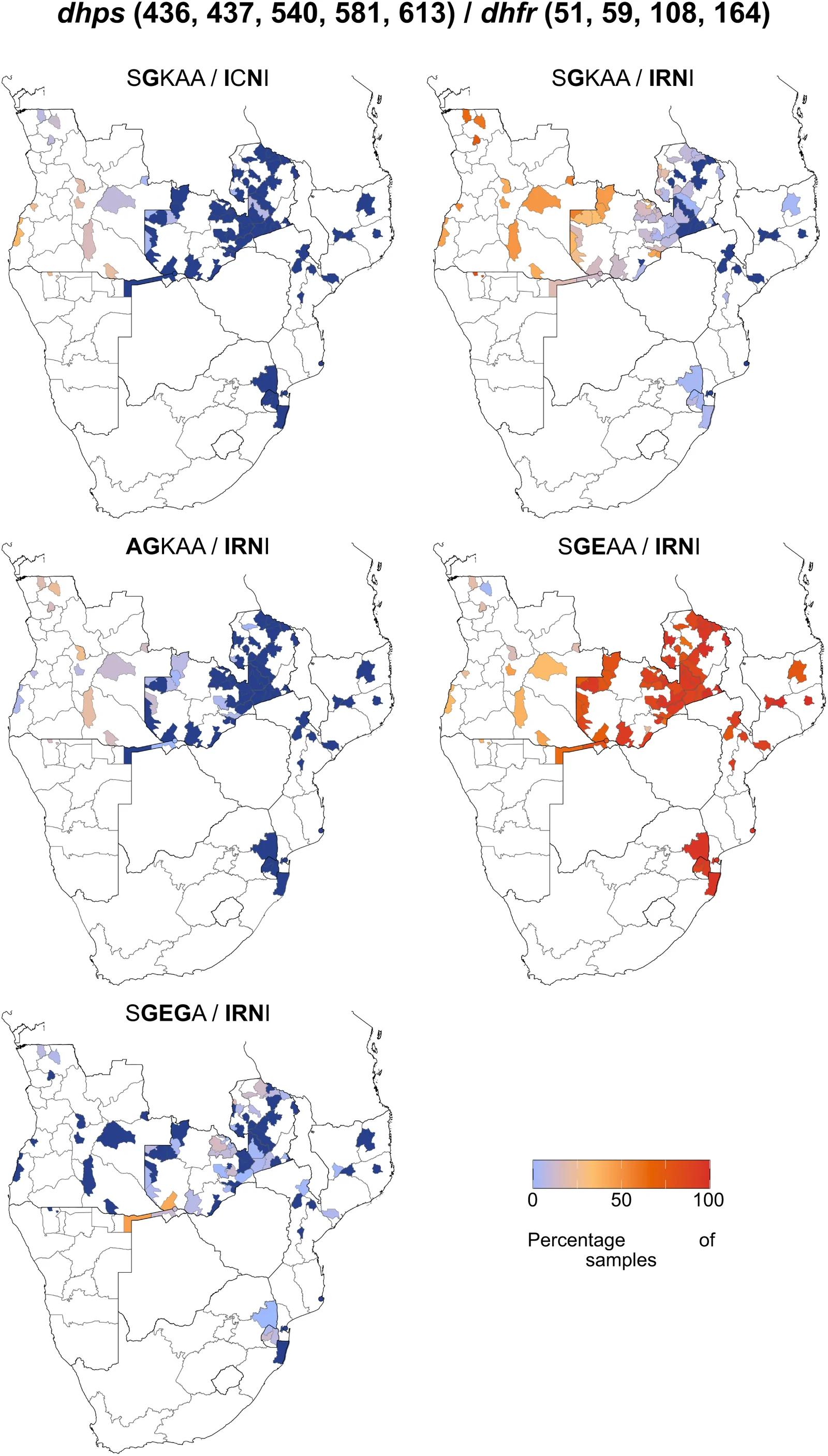
Proportion of samples carrying different dihydropteroate synthase (*dhps*) and dihydrofolate reductase (*dhfr*) combined haplotypes across districts in the Elimination Eight countries participating in the Regional Genomics for Malaria in the Southern African Development Community’s Malaria Elimination Eight and GenMoz studies. Proportions are calculated as the number of samples with a haplotype (pure or mixed) divided by the total number of genotyped infections. Only haplotypes detected in >5% of samples from any country are shown. Dark blue shading indicates the absence of a marker. Haplotypes are shown with wild-type alleles in regular font and mutant alleles in bold.

In contrast to the *dfhr* triple mutation, the *dhps* double mutation (*dhps* A437G and K540E), which is associated with sulfadoxine resistance in southern Africa, exhibited marked regional heterogeneity.^8^ This mutation ranged from less than 40% of samples in Angola to near fixation in South Africa, generally occurring on a haplotype carrying wild-type *dhps* S436, A581, and A613 alleles (Figure 3; Supplemental Figure 2; Supplemental Table 3). The second most commonly observed haplotype, *dhps* A437G/K540, was most frequently detected in northwestern Angola but was rarer in the southeastern countries. The *dhps* triple mutation, A437G/K540E/A581G, which is associated with higher levels of sulfadoxine resistance, was observed in 11.2% of samples from Namibia, 6.1% of samples from Eswatini, and more than 10% of samples in several districts in Zambia and in Mbanza-Congo District, Zaire Province, northern Angola.^39^ The *dhps* A613S mutation was rare, detected in three samples on the A437G/K540E haplotype, with one sample from Mozambique, one from South Africa, and one from Zambia. It was also observed in four samples from Eswatini on a haplotype carrying wild-type *dhps* K540 along with mutant *dhps* I431V, A437G, and A581G alleles.

### Mutations in the *crt* gene.

The distribution of mutations in the *crt* gene was also heterogeneous across the study area (Figure 4; Supplemental Figure 3; Supplemental Table 4). The *crt* K76T mutation, which is associated with chloroquine resistance, was rare in most countries except for Angola, where it was found in 19.1% of samples on a haplotype carrying *crt* M74I and N75E, with wild-type *crt* C72 and V73.^40^ A northwest-to-southeast *crt* K76T mutation gradient was observed, with the mutation most frequent in the northern Angola provinces of Zaire and Uíge (>50% of samples) and decreasing toward the shared borders with Namibia and Zambia (<10%; Figure 4). Similar regional patterns were observed for *crt* A220S, which is also associated with chloroquine resistance, and *crt* I356T, which is associated with reduced susceptibility to quinine and increased susceptibility to mefloquine (Supplemental Table 4).^41^ Although generally present at low levels (<3%), the *crt* F48L mutation was detected in all countries except Namibia (Supplemental Table 4).

**Figure 4.**
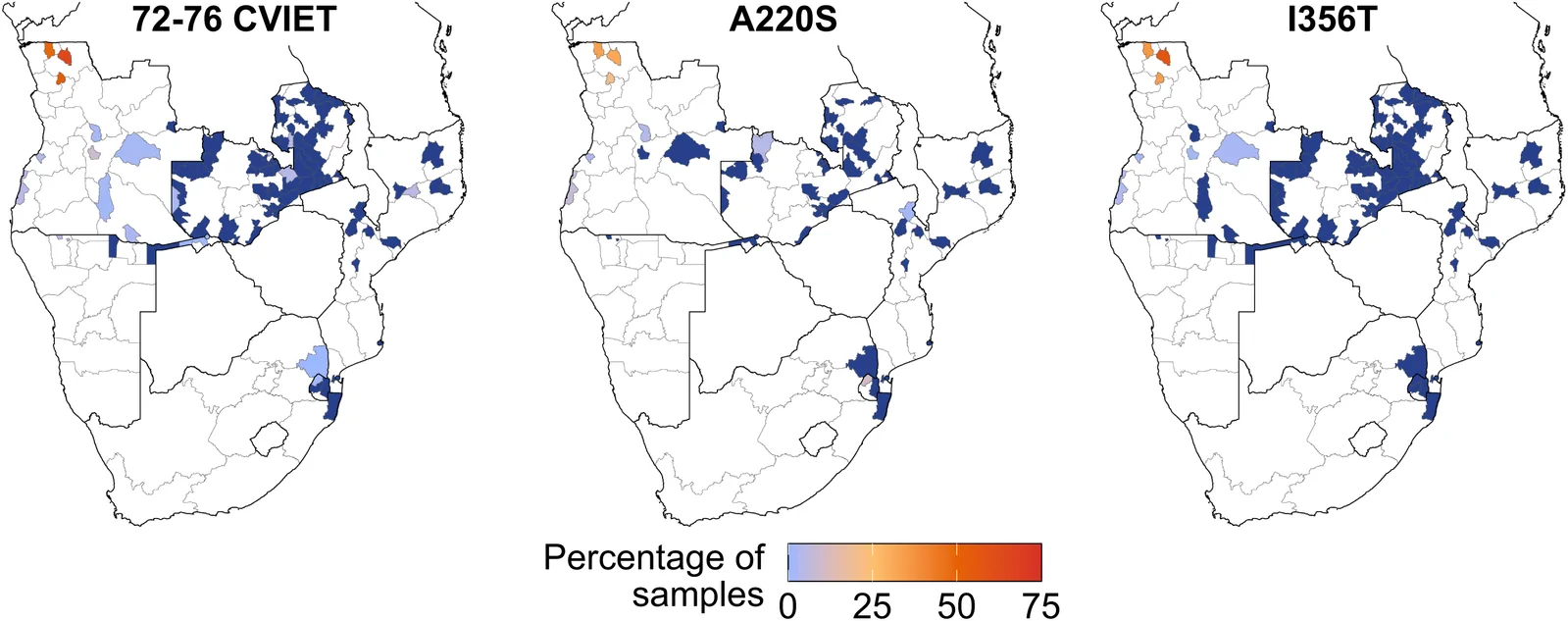
Proportion of samples with mutations in the chloroquine resistance transporter gene across districts in the Elimination Eight countries participating in the Regional Genomics for Malaria in the Southern African Development Community’s Malaria Elimination Eight and GenMoz studies. Proportions are calculated as the number of samples with a haplotype (pure or mixed) divided by the total number of genotyped infections. Dark blue shading indicates absence of a marker.

### Mutations in the *mdr1* gene.

The *mdr1* N86Y mutation, which modulates chloroquine resistance and is associated with susceptibility to lumefantrine, was rarely detected, as was the D1246Y mutation (Table 2; Supplemental Table 5).^42^ In contrast, the *mdr1* Y184F mutation was detected in at least 50% of samples from each participating country, with the highest frequencies in Eswatini, Mozambique, and South Africa. Among the *mdr*1 haplotypes detected, the wild-type *mdr1* NYD (N86, Y184, and D1246) haplotype was the most prevalent, followed by the single-mutant haplotype NFD (N86, Y184F, and D1246).

Other mutations listed in the WHO compendium of molecular markers for antimalarial drug resistance were either not detected (*crt* C72S, T93S, H97Y, C101F, I218F, N326D, M343L, C350R, G353V, and I356L; *coronin* G50E, R100K, and E107V; *dhps* A16V, *mdr1* S1034C, N1042D) or were not assessed (*crt* G367C, polymorphisms in cytochrome b gene and *mdr1* copy number variations).^43^ The *exo* E415G mutation, which is associated with piperaquine resistance, was detected in a single sample from Zambia’s Muchinga Province.^44^ Polymorphisms hypothesized to constitute a genetic background for ART-R were uncommon, except for the *mdr2* I492V mutation, which occurred at district-level proportions ranging from 15% to 80%, with lower values in southern countries. The *mdr2* T484I mutation was detected in single samples from Zambia and South Africa, whereas the *px1* C1484F and *fd* D193Y alleles were each detected in one sample from Zambia and South Africa, respectively. The *arps10* V127M and D128H mutations and *crt* N326S allele were not detected, whereas the presence of the *pph* V1157L mutation was not evaluated.^45^

## DISCUSSION

By integrating molecular surveillance data from six southern African countries generated through harmonized sampling and analytical approaches, we provide the first regional synthesis of antimalarial drug resistance marker distribution across southern Africa. The findings have direct implications for case management and chemoprevention policies and inform priority areas for research and TESs.

Although validated *kelch13* ART-R markers remained rare across the study region, never exceeding 1.3% in any country, pronounced spatial heterogeneity at the subnational level was detected. Validated and candidate ART-R markers, as well as uncharacterized *kelch13* mutations, were concentrated in districts along the shared Angola–Namibia–Zambia border and in central Zambia. Notably, the candidate ART-R marker *kelch13* P441L exceeded 30% in districts in eastern Namibia and Zambia’s Central and Western Provinces. This mutation has now been reported across multiple eastern African countries with evidence of positive selection, although its clinical relevance in an African setting requires further investigation.^46^^–^^48^ It is also unclear whether the southern African lineages share a common ancestor with eastern African lineages or arose independently.

Outside the Angola–Namibia–Zambia hotspot, validated ART-R markers were rare. A small cluster of genetically related *kelch13* P553L infections comprising four cases from Eswatini and a single case from South Africa were detected, with travel histories suggesting importation from Mozambique. However, this mutation was not detected in Mozambican samples from the preceding transmission season. This mutation has not been detected in South Africa since, so it is possible that this mutation alone offered no selective advantage in southern Africa.

The epidemiological, ecological, and biological factors underpinning ART-R emergence and spread are not fully understood. Drug pressure in low-transmission settings, where host immunity is lower and parasite competition is reduced, has been hypothesized to facilitate the emergence of resistance.^49^ However, in the present regional analysis, *kelch13* mutations were observed across all transmission strata. The Angola–Namibia–Zambia hotspot is characterized by highly heterogeneous and seasonal transmission intensities, with very low-transmission districts prone to malaria outbreaks bordering high-transmission areas.

Southern Africa is characterized by highly mobile populations, whose cross-border mobility shapes malaria transmission and parasite connectivity across the region.^50^ These populations often face barriers to accessing appropriate care and malaria prevention, which, in conjunction with occupational or behavioral exposure to infectious mosquitoes, facilitates parasite movement between areas.^51^^,^^52^ Such importation may have contributed to the geographic clustering of *kelch13* mutations in Namibia and Zambia, where established mobility corridors exist. Although population movement may help to explain the distribution of resistance markers, it is insufficient to explain the emergence of *kelch13* mutations. Community- or household-level targeted MDA with artemisinin-based regimens has been trialed in both countries in the past decade.^29^^,^^30^ Whether these interventions contributed to the *kelch13* patterns observed cannot be determined from the present data. However, other southern African regions with similarly low transmission intensities, including eliminating districts in Eswatini, Mozambique, and South Africa, exhibited only limited evidence of validated ART-R markers, despite Eswatini also trialing artemisinin-based focal MDA.^28^ This suggests that neither drug pressure nor malaria importation alone adequately explain the *kelch13* P441L hotspots detected in the RegGenE8 study. Emergence of antimalarial resistance markers is likely influenced by differences in parasite genetic background, access to care, antimalarial drug stewardship, and other contextual factors. Deep sequencing of archived samples across multiple transmission seasons, integrated with human mobility and transmission intensity data, would enable reconstruction of the evolutionary history of these mutations in this region and the relative contributions of parasite spread and local emergence.

The wild-type *mdr1* N86 allele was at or nearing fixation across all six countries. The clinical impact of this allele on lumefantrine efficacy remains unresolved. A meta-analysis revealed an association with artemether–lumefantrine treatment failures, whereas in vitro investigations revealed only modest changes in lumefantrine susceptibility.^53^^,^^54^ A recently described haplotype in the *px1* gene associated with decreased lumefantrine susceptibility was not evaluated here.^55^ No mutations associated with resistance to piperaquine or amodiaquine, other ACT partner drugs, were detected. Copy number variation markers associated with piperaquine and lumefantrine resistance were not assessed.^43^

Encouragingly, efficacies greater than 90% have been reported for artemether–lumefantrine, which is the preferred ACT in all six study countries, across Mozambique, and in Zambia (National Malaria Elimination Centre (NMEC), personal communication).^56^ However, in three Angolan provinces Bengo, Lunda Sul, and Zaire, efficacies below the WHO 90% threshold have been observed.^57^^,^^58^ Interestingly, no validated or candidate ART-R markers were detected in Zaire Province in this or previous studies revealing reduced efficacy, suggesting that treatment failures in these areas are not driven primarily by ART-R. The recent observation of *mdr1* gene duplications in Angolan treatment failures points toward reduced lumefantrine susceptibility as a potential contributor.^58^

In the districts where *kelch13* mutations were identified, parasites predominantly carried the *mdr1* N86 wild-type allele, which may be associated with reduced lumefantrine susceptibility. Although clinical failures have not yet been widely reported in these areas, the combination of emerging *kelch13* variants and a potentially lumefantrine-tolerant genetic background warrants close monitoring. Erosion of partner drug efficacy may precede or potentiate ACT resistance.^59^ However, the low prevalence of ART-R markers combined with high artemether–lumefantrine efficacy data suggests that there is currently no urgent concern for ACT efficacy across the six participating countries outside the localized areas reporting reduced artemether–lumefantrine efficacy or elevated *kelch13* mutation frequencies.

Although SP resistance does not directly threaten first-line ACT efficacy, its regional distribution reinforces a broader picture of a resistance landscape shaped by drug pressure gradients and cross-border parasite movement. The prevalence of the *dhfr*-*dhps* quintuple mutation was markedly heterogeneous across the region, with Angola and northwestern Namibia exhibiting substantially lower SP resistance prevalence than the southeastern countries, where it approached fixation. Notably, the observed *dhfr*-*dhps* quintuple mutation distribution does not correspond with contemporary SP use. Eswatini, Namibia, and South Africa do not currently use SP for treatment or chemoprevention, yet quintuple-mutant prevalence in these countries was high. This suggests that the current mutation distribution more likely reflects historical and regional drug pressure gradients than present-day national drug pressure and policy. This pattern positions Angola and Namibia at the transition zone within the continental west-to-east gradient of low to high *dhps* K540E prevalence.^60^ The quintuple haplotype combined with the *dhps* A581G mutation, which is associated with reduced SP-IPT in pregnancy efficacy, exceeded 5% in Eswatini, Namibia, Angola (Zaire Province), and several Zambian districts.^61^^,^^62^ Because SP is not used for treatment or chemoprevention in Eswatini and Namibia, it is unclear what drives the selection of the *dhps* A581G mutation in these countries. National malaria programs implementing SP-IPT in pregnancy should consider chemo-preventive efficacy studies and potential regimen changes in areas where this sextuple haplotype is prevalent.

Several study limitations warrant consideration. Sampling for most countries covered a single transmission season, limiting the ability to assess temporal trends in resistance marker selection at a regional level. The mismatch in the GenMoz and RegGenE8 sampling periods (Mozambique in 2022 versus most other countries in 2023) may have obscured cross-border resistance signals; contemporaneous sampling across all participating countries should be a priority in future regional studies. Both Botswana and Zimbabwe declined to participate in RegGenE8, creating a corridor of uncertainty between the Angola–Namibia–Zambia block and the Eswatini–Mozambique–South Africa block. Whether *kelch13* mutations have spread into these countries, and how far south they may be present, remains unknown. To the best of the authors’ knowledge, no recent TES data are available from Zimbabwe, further limiting the ability to assess ACT efficacy across this corridor. Generating resistance and efficacy data for both countries is an urgent priority. Finally, only validated markers of resistance were assessed using an amplicon sequencing approach in the studies. By design, this targeted strategy cannot be used to detect signatures of selection at novel or unconventional genomic regions arising from drug pressure. Whole-genome sequencing of recrudescent parasites, and more broadly across surveyed populations, remains the most direct approach to identifying such signatures and characterizing novel markers with clinical relevance.^63^ Amplicon-based surveillance assays should be routinely updated as novel markers are identified and validated.^55^^,^^64^

## CONCLUSION

The molecular landscape described here provides valuable insight into antimalarial drug resistance marker distribution across six southern African countries and can be used to guide preparedness planning for resistance prevention and containment. In districts where *kelch13* mutation frequencies are rising, ensuring effective treatment availability is paramount. Modeling studies have indicated that multiple first-line therapy strategies and triple ACTs are among the most effective single policies for slowing the spread of ART-R and reducing treatment failures.^65^^,^^66^ Rapid data sharing between neighboring national malaria programs and the generation of maps that integrate parasite genomic and human mobility data with transmission intensity will support the identification of districts most at risk, enabling prospective targeting of preventive interventions. As the region moves toward elimination, sustaining and expanding the molecular surveillance infrastructure established by RegGenE8 and GenMoz to include all E8 countries will be essential to protect treatment efficacy and support elimination goals across southern Africa.

## Supplemental Materials

10.4269/ajtmh.26-0299 Supplemental Materials

## References

[1] Gao L Shi Q Liu Z Li Z Dong X, 2023. Impact of the COVID-19 pandemic on malaria control in Africa: A preliminary analysis. Trop Med Infect Dis 8(1): 67. 10.3390/tropicalmed8010067 PMC9863638 36668974

[2] World Health Organization, 2024. *World Malaria Report 2024: Addressing Inequity in the Global Malaria Response*. Available at: https://www.who.int/teams/global-malaria-programme/reports/world-malaria-report-2024. Accessed September 17, 2026.

[3] World Health Organization, 2025. *World Malaria Report 2025: Addressing the Threat of Antimalarial Drug Resistance*. Available at: https://www.who.int/teams/global-malaria-programme/reports/world-malaria-report-2025. Accessed September 17, 2026.

[4] Rosenthal PJ Asua V Bailey JA Conrad MD Ishengoma DS Kamya MR Rasmussen C Tadesse FG Uwimana A Fidock DA, 2024. The emergence of artemisinin partial resistance in Africa: How do we respond? Lancet Infect Dis 24(9): e591–e600. 10.1016/S1473-3099(24)00141-5 PMC12954456 38552654

[5] Trape JF, 2001. The public health impact of chloroquine resistance in Africa. Am J Trop Med Hyg 64(1–2 Suppl): 12–17. 10.4269/ajtmh.2001.64.12 11425173

[6] Raman J, , 2016. Reviewing South Africa’s malaria elimination strategy (2012–2018): Progress, challenges and priorities. Malar J 15(1): 438. 10.1186/s12936-016-1497-x PMC5002155 27567642

[7] Payne D, 1987. Spread of chloroquine resistance in *Plasmodium falciparum*. Parasitol Today 3(8): 241–246. 10.1016/0169-4758(87)90147-5 15462966

[8] Roper C Pearce R Nair S Sharp B Nosten F Anderson T, 2004. Intercontinental spread of pyrimethamine-resistant malaria. Science 305(5687): 1124. 10.1126/science.1098876 15326348

[9] Wootton JC Feng X Ferdig MT Cooper RA Mu J Baruch DI Magill AJ Su X, 2002. Genetic diversity and chloroquine selective sweeps in *Plasmodium falciparum*. Nature 418(6895): 320–323. 10.1038/nature00813 12124623

[10] Bhatt S, , 2015. The effect of malaria control on *Plasmodium falciparum* in Africa between 2000 and 2015. Nature 526(7572): 207–211. 10.1038/nature15535 PMC4820050 26375008

[11] World Health Organization, 2021. *Global Technical Strategy for Malaria 2016–2030, 2021 Update*. Available at: https://www.who.int/publications/i/item/9789240031357. Accessed September 17, 2026.

[12] World Health Organization, 2022. *Strategy to Respond to Antimalarial Drug Resistance in Africa*. Available at: https://www.who.int/publications/i/item/9789240060265. Accessed September 17, 2026.

[13] Ishengoma DS, , 2024. Urgent action is needed to confront artemisinin partial resistance in African malaria parasites. Nat Med 30(7): 1807–1808. 10.1038/d41591-024-00028-y PMC12989122 38710833

[14] Balmer AJ White NF Ünlü ES Lee C Pearson RD Almagro-Garcia J Ariani C, 2025. Understanding the global rise of artemisinin resistance: Insights from over 100,000 *Plasmodium falciparum* samples. Elife 14: e105544. 10.7554/eLife.105544 PMC12490857 41037007

[15] World Health Organization, 2026. *Malaria Threat Map*. Available at: https://apps.who.int/malaria/maps/threats/. Accessed September 17, 2026.

[16] Raman J Fakudze P Sikaala CH Chimumbwa J Moonasar D, 2021. Eliminating malaria from the margins of transmission in Southern Africa through the Elimination 8 Initiative. Transact Royal Soc South Africa 76(2): 137–145.

[17] Mayor A, , 2022. Prospective surveillance study to detect antimalarial drug resistance, gene deletions of diagnostic relevance and genetic diversity of *Plasmodium falciparum* in Mozambique: Protocol. BMJ Open 12(7): e063456. 10.1136/bmjopen-2022-063456 PMC9274532 35820756

[18] Malaria Atlas Project, 2026. *Malaria Atlas Project Pf Incidence Rate 2023*. Available at: https://data.malariaatlas.org/trends?year=2023&metricGroup=Malaria&geographicLevel=admin1&metricSubcategory=Pf&metricType=rate&metricName=incidence. Accessed September 17, 2026.

[19] World Health Organization, 2017. *A Framework for Malaria Elimination*. Available at: https://www.who.int/publications/i/item/9789241511988. Accessed September 17, 2026.

[20] João MF, , 2026. Geographical heterogeneity in antimalarial resistance markers by genomic surveillance in Angola, 2023. Am J Trop Med Hyg 115(Suppl 5): 19–30. 10.4269/ajtmh.25-0226 PMC13736874 41843937

[21] Nhlengethwa N, , 2026. Genomic surveillance reveals clusters of *Plasmodium falciparum* antimalarial resistance markers in Eswatini, a low-transmission setting. Am J Trop Med Hyg 115(Suppl 5): 31–43. 10.4269/ajtmh.25-0487 PMC13737250 42155405

[22] Eloff L, , 2026. High prevalence of molecular markers associated with artemisinin, sulphadoxine and pyrimethamine resistance in Northern Namibia. Am J Trop Med 115(Suppl 5): 44–52. 10.4269/ajtmh.24-0870 PMC13736803 40744004

[23] Raman J, , 2026. Very low prevalence of validated *kelch13* mutations and absence of *Histidine-Rich Protein* 2/3 double gene deletions in South African malaria-eliminating districts (2022–2024). Am J Trop Med Hyg 115(Suppl 5): 53–64. 10.4269/ajtmh.25-0204 PMC13737031 41130222

[24] Aranda-Díaz A, , 2026. *Plasmodium falciparum* genomic surveillance reveals a diversity of *Kelch13* mutations in Zambia. Am J Trop Med Hyg 115(Suppl 5): 65–76. 10.4269/ajtmh.25-0110 PMC13736986 40744006

[25] Boene S, , 2025. Antimalarial drug resistance and population structure of *Plasmodium falciparum* in Mozambique using genomic surveillance at health facilities in 2021 and 2022. Sci Rep 15(1): 29335. 10.1038/s41598-025-02166-w PMC12340125 40790052

[26] Zambia National Malaria Elimination Centre, 2022. *National Malaria Elimination Strategic Plan 2022–2026*. Available at: https://mesamalaria.org/resource-hub/national-malaria-elimination-strategic-plan-nmesp-of-zambia-2022-2026/. Accessed September 17, 2026.

[27] Canana N Tarquino IAP Enosse S Baker K Rodrigues M Rassi C Chauhan AS Nnaji C Candrinho B Maffioli EM, 2025. Seasonal malaria chemoprevention in northern Mozambique: A cost-effectiveness analysis. Malar J 24(1): 159. 10.1186/s12936-025-05401-x PMC12096524 40399941

[28] Vilakati S, , 2021. Effectiveness and safety of reactive focal mass drug administration (rfMDA) using dihydroartemisinin–piperaquine to reduce malaria transmission in the very low-endemic setting of Eswatini: A pragmatic cluster randomised controlled trial. BMJ Glob Health 6(6): e005021. 10.1136/bmjgh-2021-005021 PMC8246301 34193475

[29] Eisele TP, , 2020. Impact of four rounds of mass drug administration with dihydroartemisinin–piperaquine implemented in Southern Province, Zambia. Am J Trop Med Hyg 103(Suppl 2): 7–18. 10.4269/ajtmh.19-0659 PMC7416977 32618247

[30] Hsiang MS, , 2020. Effectiveness of reactive focal mass drug administration and reactive focal vector control to reduce malaria transmission in the low malaria-endemic setting of Namibia: A cluster-randomised controlled, open-label, two-by-two factorial design trial. Lancet 395(10233): 1361–1373. 10.1016/S0140-6736(20)30470-0 PMC7184675 32334702

[31] Aranda-Díaz A, , 2025. Sensitive and modular amplicon sequencing of *Plasmodium falciparum* diversity and resistance for research and public health. Sci Rep 15(1): 10737. 10.1038/s41598-025-94716-5 PMC11953298 40155691

[32] Teyssier NB Chen A Duarte EM Sit R Greenhouse B Tessema SK, 2021. Optimization of whole-genome sequencing of *Plasmodium falciparum* from low-density dried blood spot samples. Malar J 20(1): 116. 10.1186/s12936-021-03630-4 PMC7912882 33637093

[33] Hofmann N Mwingira F Shekalaghe S Robinson LJ Mueller I Felger I, 2015. Ultra-sensitive detection of *Plasmodium falciparum* by amplification of multi-copy subtelomeric targets. PLoS Med 12(3): e1001788. 10.1371/journal.pmed.1001788 PMC4348198 25734259

[34] Mayor A, , 2009. Sub-microscopic infections and long-term recrudescence of *Plasmodium falciparum* in Mozambican pregnant women. Malar J 8: 9. 10.1186/1475-2875-8-9 PMC2633011 19134201

[35] Hathaway NJ, , 2025. *The Portable Microhaplotype Object and Tools*. Available at: https://www.biorxiv.org/content/10.64898/2025.12.10.693568v1. Accessed September 17, 2026.

[36] Ménard D, , 2016. A worldwide map of *Plasmodium falciparum* K13-propeller polymorphisms. N Engl J Med 374(25): 2453–2464. 10.1056/NEJMoa1513137 PMC4955562 27332904

[37] World Health Organization, 2020. *Report on Antimalarial Drug Efficacy, Resistance and Response: 10 Years of Surveillance (2010–2019)*. Available at: https://www.who.int/publications/i/item/9789240012813. Accessed September 22, 2026.

[38] HastingsMD, et al. 2002. Highly pyrimethamine-resistant alleles of dihydrofolate reductase in isolates of *Plasmodium falciparum* from Tanzania. *Trans R Soc Trop Med Hyg* 96(6):674–676. 10.1016/s0035-9203(02)90349-4 12630380

[39] Gesase S, , 2009. High resistance of *Plasmodium falciparum* to sulphadoxine/pyrimethamine in Northern Tanzania and the emergence of *dhps* resistance mutation at codon 581. PLoS One 4(2): e4569. 10.1371/journal.pone.0004569 PMC2644264 19238219

[40] Djimdé A, , 2001. A molecular marker for chloroquine-resistant falciparum malaria. N Engl J Med 344(4): 257–263. 10.1056/NEJM200101253440403 11172152

[41] Foguim FT Bogreau H Gendrot M Mosnier J Fonta I Benoit N Amalvict R Madamet M Wein S Pradines B, 2020. Prevalence of mutations in the *Plasmodium falciparum* chloroquine resistance transporter, PfCRT, and association with ex vivo susceptibility to common anti-malarial drugs against African *Plasmodium falciparum* isolates. Malar J 19(1): 201. 10.1186/s12936-020-03281-x PMC7275453 32503540

[42] Rasmussen SA, , 2017. Changing antimalarial drug sensitivities in Uganda. Antimicrob Agents Chemother 61(12): e01516–e01517. 10.1128/AAC.01516-17 PMC5700361 28923866

[43] World Health Organization, 2025. *Compendium of Molecular Markers for Antimalarial Drug Resistance*. Available at: https://www.who.int/tools/compendium-of-molecular-markers-for-antimalarial-drug-resistance. Accessed September 17, 2026.

[44] Witkowski B, , 2017. A surrogate marker of piperaquine-resistant *Plasmodium falciparum* malaria: A phenotype–genotype association study. Lancet Infect Dis 17(2): 174–183. 10.1016/S1473-3099(16)30415-7 PMC5266792 27818097

[45] Miotto O, , 2015. Genetic architecture of artemisinin-resistant *Plasmodium falciparum*. Nat Genet 47(3): 226–234. 10.1038/ng.3189 PMC4545236 25599401

[46] Conrad MD, , 2023. Evolution of partial resistance to artemisinins in malaria parasites in Uganda. N Engl J Med 389(8): 722–732. 10.1056/NEJMoa2211803 PMC10513755 37611122

[47] Meier-Scherling CPG Watson OJ Asua V Ghinai I Katairo T Garg S Conrad MD Rosenthal PJ Okell LC Bailey JA, 2025. Selection of *Plasmodium falciparum kelch13* mutations in Uganda in comparison with southeast Asia: A modelling study. Lancet Microbe 6(5): 101027. 10.1016/j.lanmic.2024.101027 PMC12128186 40112841

[48] Connelly SV, , 2026. Artemisinin partial resistance mutations in Zanzibar and Tanzania suggest regional spread and African origins, 2023. J Infect Dis 233(2): e491–e495. 10.1093/infdis/jiaf431 PMC13017645 40802860

[49] Masserey T Lee T Golumbeanu M Shattock AJ Kelly SL Hastings IM Penny MA, 2022. The influence of biological, epidemiological, and treatment factors on the establishment and spread of drug-resistant *Plasmodium falciparum*. Elife 11: e77634. 10.7554/eLife.77634 PMC9262398 35796430

[50] Chisenga M, , 2023. The provision of malaria services in border districts of four countries in Southern Africa: Results from a cross-sectional community assessment of malaria border health posts. Malar J 22(1): 318. 10.1186/s12936-023-04687-z PMC10588083 37864245

[51] Jacobson JO Cueto C Smith JL Hwang J Gosling R Bennett A, 2017. Surveillance and response for high-risk populations: What can malaria elimination programmes learn from the experience of HIV? Malar J 16(1): 33. 10.1186/s12936-017-1679-1 PMC5241929 28100237

[52] Lynch C Roper C, 2011. The transit phase of migration: Circulation of malaria and its multidrug-resistant forms in Africa. PLoS Med 8(5): e1001040. 10.1371/journal.pmed.1001040 PMC3104977 21655316

[53] Venkatesan M, , 2014. Polymorphisms in *Plasmodium falciparum* chloroquine resistance transporter and multidrug resistance 1 genes: Parasite risk factors that affect treatment outcomes for *P. falciparum* malaria after artemether-lumefantrine and artesunate-amodiaquine. Am J Trop Med Hyg 91(4): 833–843. 10.4269/ajtmh.14-0031 PMC4183414 25048375

[54] Tumwebaze PK, , 2021. Drug susceptibility of *Plasmodium falciparum* in eastern Uganda: A longitudinal phenotypic and genotypic study. Lancet Microbe 2(9): e441–e449. 10.1016/s2666-5247(21)00085-9 PMC8454895 34553183

[55] Niaré K, , 2025. *A Novel Locus Associated with Decreased Susceptibility of Plasmodium falciparum to Lumefantrine and Dihydroartemisinin Has Emerged and Spread in Uganda*. Available at: https://www.biorxiv.org/content/10.1101/2025.07.30.667738v2. Accessed September 17, 2026.

[56] Nhama A, , 2025. Efficacy of artemether-lumefantrine, artesunate-amodiaquine, dihydroartemisinin-piperaquine and artesunate-pyronaridine for the treatment of uncomplicated *Plasmodium falciparum* malaria in Mozambique, 2022. Malar J 24(1): 231. 10.1186/s12936-025-05473-9 PMC12257838 40660279

[57] Dimbu PR, , 2024. Therapeutic response to four artemisinin-based combination therapies in Angola, 2021. Antimicrob Agents Chemother 68(4): e0152523. 10.1128/aac.01525-23 PMC10989004 38421163

[58] Fançony C, , 2025. Artemether-lumefantrine treatment selects *Plasmodium falciparum* multidrug resistance 1 (*pfmdr1*) increased copy number among African malaria infections. J Infect Dis 231(6): e1119–e1128. 10.1093/infdis/jiaf155 PMC12247823 40138574

[59] Rosenthal PJ Asua V Conrad MD, 2024. Emergence, transmission dynamics and mechanisms of artemisinin partial resistance in malaria parasites in Africa. Nat Rev Microbiol 22(6): 373–384. 10.1038/s41579-024-01008-2 38321292

[60] Flegg JA Humphreys GS Montanez B Strickland T Jacome-Meza ZJ Barnes KI Raman J Guerin PJ Hopkins Sibley C Dahlström Otienoburu S, 2022. Spatiotemporal spread of *Plasmodium falciparum* mutations for resistance to sulfadoxine-pyrimethamine across Africa, 1990–2020. PLoS Comput Biol 18(8): e1010317. 10.1371/journal.pcbi.1010317 PMC9371298 35951528

[61] van Eijk AM Stepniewska K Khairallah C Rodriguez E Ahn J Gutman JR Ter Kuile FO, 2025. The impact of sulfadoxine–pyrimethamine resistance on the effectiveness of intermittent preventive treatment for the prevention of malaria in pregnancy in Africa: An updated systematic review and meta-analysis. Lancet Infect Dis 25(12): 1336–1346. 10.1016/S1473-3099(25)00219-1 PMC12743599 40675171

[62] Chico RM Cano J Ariti C Collier TJ Chandramohan D Roper C Greenwood B, 2015. Influence of malaria transmission intensity and the 581G mutation on the efficacy of intermittent preventive treatment in pregnancy: Systematic review and meta-analysis. Trop Med Int Health 20(12): 1621–1633. 10.1111/tmi.12595 26325263

[63] Ariey F, , 2014. A molecular marker of artemisinin-resistant *Plasmodium falciparum* malaria. Nature 505(7481): 50–55. 10.1038/nature12876 PMC5007947 24352242

[64] Mwenda M, , 2026. *Emergence and Spread of Artemisinin-Resistant Malaria in Zambia*. Available at: https://www.medrxiv.org/content/10.64898/2026.06.04.26354343v1.full. Accessed September 22, 2026.

[65] Zupko RJ Nguyen TD Ngabonziza JCS Kabera M Li H Tran TN-A Tran KT Uwimana A Boni MF, 2023. Modeling policy interventions for slowing the spread of artemisinin-resistant pfkelch R561H mutations in Rwanda. Nat Med 29(11): 2775–2784. 10.1038/s41591-023-02551-w PMC10667088 37735560

[66] Nguyen TD Gao B Amaratunga C Dhorda M Tran TN-A White NJ Dondorp AM Boni MF Aguas R, 2023. Preventing antimalarial drug resistance with triple artemisinin-based combination therapies. Nat Commun 14(1): 4568. 10.1038/s41467-023-39914-3 PMC10387089 37516752

