# Supplementary material for: Geographical Variation in Antimalarial Drug Resistance Marker Prevalence across the Southern African Elimination Eight Region
Source: Am J Trop Med Hyg. 2026 Oct 6;115(5 Suppl):5–18. doi: 10.4269/ajtmh.26-0299 (PMC13675676; doi:10.4269/ajtmh.26-0299)
Supplement: Supplementary file 1 [file tpmd260299.SD1.pdf]

Supplement for:

Geographical Variation in Antimalarial Drug Resistance Marker Prevalence Across the Southern African Elimination 8 Region

Authors:

Jaishree Raman*[1,2,3], Andrés Aranda-Díaz*[4,5,6#], Maxwell Mabona[1], Mukosha Chisenga[7], Maria Florinda João[8], Domingos Jandondo[9], Pedro Rafael Dimbu[8], Nomcebo Nhlengethwa[10], Sabelo V. Dlamini[11], Lydia Eloff[12,13], Stark Katokele[14], Davis R. Mumbengegwi[13], Qedusizi Nyawo[15], Mbavhalelo Shandukani[16], Sydney Mwanza[17], Moonga Hawela[18], Simone Boene[6,19,20], Arlindo Chidimatembue[19], Bernardete Rafael[21], Eduard Rovira-Vallbona[6], Brighton Mangena[7], Sonja B. Lauterbach[1], Takalani I. Makhanthisa[1], Hazel Gwarinda[1], Blaženka D Letinić[1], Faith De Amaral[4], Isobel Routledge[5], Beatriz Arregui-Gallego[6], Mishalan Moodley[22], Jonathan Featherstone[22], Power B. Tshikae[15], Arshad Ismail[22], Jose Franco Martins[8], Quinton Dlamini[10], Baltazar Candrinho[21], Petrina Uusiku[14], Ednah Baloyi[16], Busiku Hamainza[18], Alfredo Mayor[6,19,20,23,24], Bryan Greenhouse[4], Amy Wesolowski[25], Chadwick Sikaala[7], John Chimumbwa[$7], Jennifer L. Smith[$5,26]

Affiliations:

[1] Laboratory for Antimalarial Resistance Monitoring and Malaria Operational Research, Centre of Emerging Zoonotic and Parasitic Diseases, National Institute for Communicable Diseases, Johannesburg, South Africa
[2] Wits Research Institute for Malaria, University of Witwatersrand, Johannesburg, South Africa
[3] University of Pretoria Institute for Sustainable Malaria Control, University of Pretoria, Pretoria, South Africa
[4] EPPIcenter Research Program, Department of Medicine, University of California, San Francisco, United States of America
[5] Malaria Elimination Initiative, Global Health Group, University of California, San Francisco, United States of America
[6] ISGlobal, Barcelona, Spain
[7] SADC Malaria Elimination Eight Secretariat, Windhoek, Namibia
[8] Programa Nacional do Controlo da Malária, Luanda, Angola
[9] Instituto Nacional de Investigação em Saúde, Luanda, Angola
[10] National Malaria Programme, Manzini, Eswatini
[11] Department of Environmental Health Sciences, University of Eswatini, Mbabane, Eswatini
[12] Department of Biochemistry, Microbiology, and Biotechnology, School of Science, University of Namibia, Windhoek, Namibia
[13] Malaria Operational Research Program, Multidisciplinary Research Services, University of Namibia, Windhoek, Namibia
[14] National Vector-Borne Disease Control Program, Ministry of Health and Social Services, Windhoek, Namibia
[15] KwaZulu-Natal Provincial Malaria Control Program, Jozini, KwaZulu-Natal, South Africa
[16] National Department of Health, Pretoria, South Africa
[17] National Health Research and Training Institute, Ndola, Zambia
[18] National Malaria Elimination Centre, Ministry of Health, Lusaka, Zambia
[19] Centro de Investigação em Saúde de Manhiça, Manhiça, Mozambique
[20] Facultat de Medicina i Ciències de la Salut, Universitat de Barcelona, Barcelona, Spain
[21] Programa Nacional do Controlo da Malária, Maputo, Mozambique
[22] Sequencing Core Facility, National Institute for Communicable Diseases, Johannesburg, South Africa
[23] Faculdade de Medicina, Universidade Eduardo Mondlane, Maputo, Mozambique
[24] Spanish Consortium for Research in Epidemiology and Public Health (CIBERESP), Madrid, Spain
[25] Department of Epidemiology Johns Hopkins University Bloomberg School of Public Health, Baltimore, Maryland, United States of America
[26] Department of Epidemiology & Biostatistics, University of California, San Francisco, United States of America

*: contributed equally
[$]: co-senior authors
[#] corresponding author: Andrés Aranda-Díaz
E-mail: andres.aranda@isglobal.org
Address: Carrer del Rosselló, 132, 5-2, 08036 Barcelona, Spain

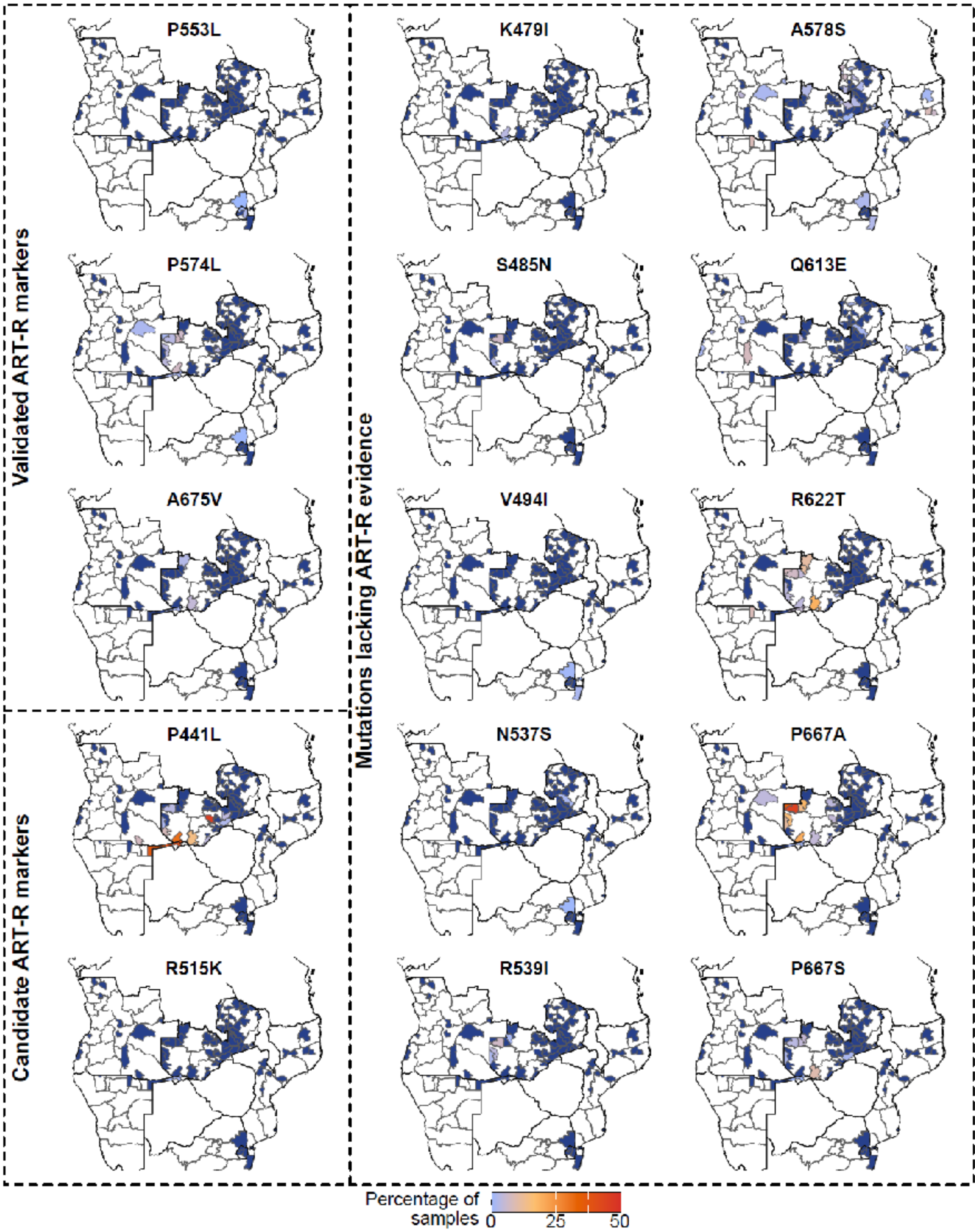

Supplemental Figure 1. Proportion of samples with non-synonymous *kelch13* mutations in the study districts from the six participating Elimination Eight countries. Proportions are calculated as the number of samples with a genotype (pure or mixed) divided by the total number of genotyped infections.

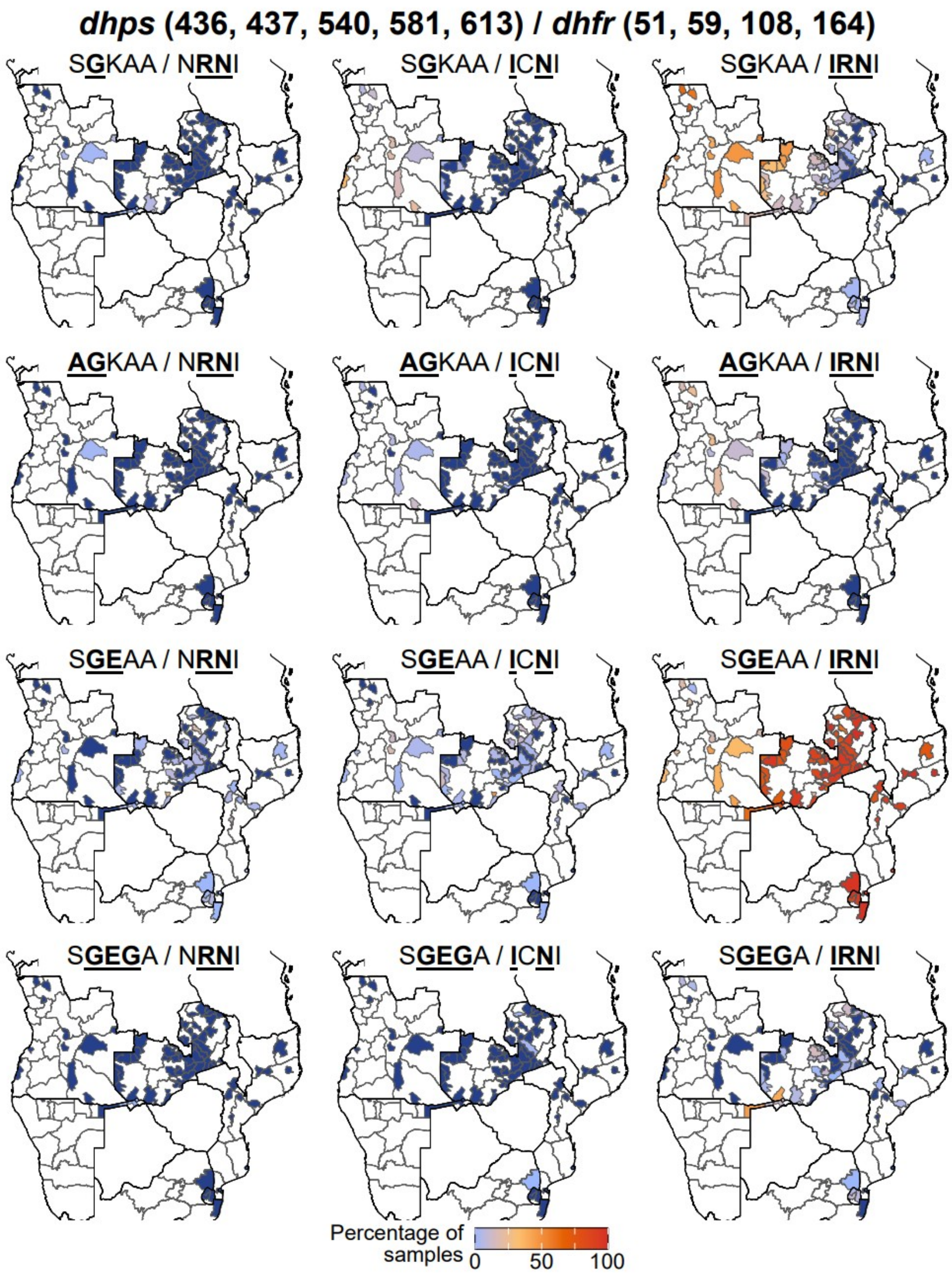

Supplemental Figure 2. Proportion of samples from the study districts in the six participating Elimination Eight countries carrying different dihydropteroate synthase (*dhps*) and dihydrofolate reductase (*dhfr*) combined haplotypes. Proportions are calculated as the number of samples with a haplotype (pure or mixed) divided by the total number of genotyped infections. Haplotypes are shown with wild-type alleles in regular font and mutant alleles in bold.

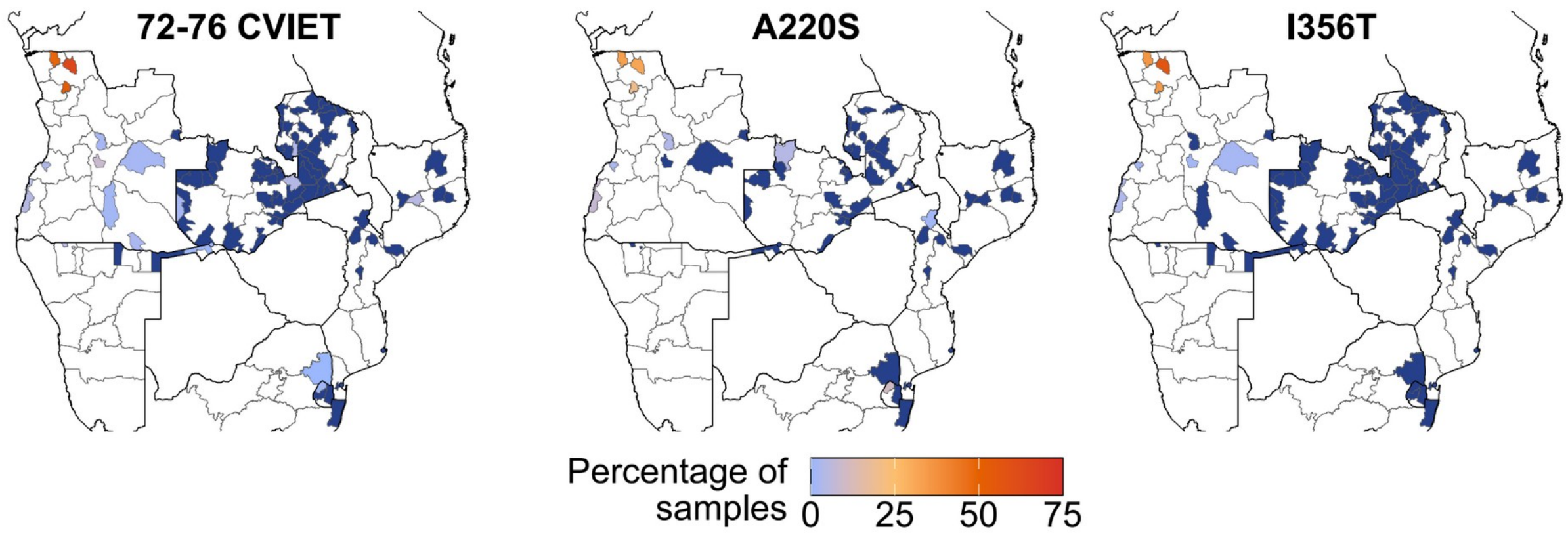

Supplemental Figure 3. Proportion of samples from the study districts in the six participating Elimination Eight countries with mutations in the chloroquine resistance transporter (*crt*) gene.

Supplemental Table 1. Per-locus depth of sequencing.

Median reads (interquartile range) and number of samples (*N*) for each amino acid and gene across study countries.

Gene	Amino acid(s)	Angola	Eswatini	Mozambique	Namibia	South Africa	Zambia
arps10	127,128	1898<br>(721-4375)<br>*N*=905	1554<br>(492-3463)<br>*N*=442	1336<br>(723-2228)<br>N=1155	8286<br>(1574-13227)<br>*N*=260	2004<br>(506-5402)<br>*N*=1455	3117<br>(1045-5898)<br>*N*=2365
coronin	50	1801<br>(602-4391)<br>*N*=883	1343<br>(446-3526)<br>*N*=435	1601<br>(948-2506)<br>*N*=1152	10756<br>(2123-19698)<br>*N*=259	2504<br>(556-7129)<br>*N*=1387	3038<br>(854-7528)<br>*N*=2304
coronin	100,107	14603<br>(4108-31305)<br>*N*=931	17542<br>(6902-40333)<br>*N*=446	2857<br>(1645-4295)<br>*N*=1162	38154<br>(6293-76162)<br>*N*=261	8678<br>(1872-30077)<br>*N*=1780	17960<br>(5996-34286)<br>*N*=2479
crt	48,52,72,73,74<br>75,76,93	6590<br>(1736-13080)<br>*N*=929	6373<br>(2696-17831)<br>*N*=446	2340<br>(1248-3688)<br>N=1162	26507<br>(3570-36094)<br>*N*=262	4863<br>(1050-15001)<br>*N*=1705	8099<br>(2691-15814)<br>*N*=2480
crt	97,101	2285<br>(588-5106)<br>*N*=897	3133<br>(1044-8500)<br>*N*=439	685<br>(383-1140)<br>*N*=1080	3115<br>(680-8802)<br>*N*=250	2094<br>(502-6010)<br>*N*=1550	2755<br>(863-5786)<br>*N*=2296
crt	145	586<br>(221-1411)<br>*N*=741	566<br>(200-1646)<br>*N*=381	551<br>(268-966)<br>*N*=1071	1978<br>(668-5960)<br>*N*=239	666<br>(214-2059)<br>*N*=1309	784<br>(257-2148)<br>*N*=2021
crt	218,220	112<br>(74-204)<br>*N*=262	123<br>(76-251)<br>*N*=99	215<br>(130-388)<br>*N*=895	186<br>(93-539)<br>*N*=146	165<br>(93-352)<br>*N*=549	143<br>(80-269)<br>*N*=773
crt	343,350,353	944<br>(298-2048)<br>*N*=826	1349<br>(456-4258)<br>*N*=431	356<br>(212-569)<br>*N*=1030	1014<br>(283-3642)<br>*N*=238	919<br>(252-2669)<br>*N*=1431	1180<br>(422-2665)<br>*N*=2177
crt	356	1585<br>(520-2872)<br>*N*=883	1876<br>(615-5406)<br>*N*=444	1226<br>(674-1886)<br>*N*=1152	3930<br>(982-8948)<br>*N*=254	1432<br>(408-4256)<br>*N*=1578	1957<br>(654-4314)<br>*N*=2379
dhfr	16,51,59	1120<br>(441-2692)<br>*N*=873	965<br>(282-1968)<br>*N*=423	1268<br>(723-1962)<br>*N*=1152	5696<br>(1122-8709)<br>*N*=257	1218<br>(314-3235)<br>*N*=1506	1694<br>(545-3512)<br>*N*=2304
dhfr	108,164	3620<br>(1268-8393)<br>*N*=913	2891<br>(967-6588)<br>*N*=441	1170<br>(618-1944)<br>*N*=1155	16357<br>(2778-25088)<br>*N*=261	3494<br>(832-10352)<br>*N*=1636	5296<br>(1728-9559)<br>*N*=2396
dhfr	185	1099<br>(412-2081)<br>*N*=889	1246<br>(525-4321)<br>*N*=436	414<br>(253-662)<br>*N*=1066	1016<br>(240-2141)<br>*N*=246	1020<br>(304-2904)<br>*N*=1559	1575<br>(597-3492)<br>*N*=2321
dhps	431,436,437	4005<br>(1396-9850)<br>*N*=917	3305<br>(1274-7945)<br>*N*=446	1577<br>(892-2581)<br>*N*=1152	11996<br>(3088-29024)<br>*N*=262	3549<br>(903-10788)<br>*N*=1661	6605<br>(2251-13023)<br>*N*=2419
dhps	540	1086<br>(444-2577)<br>*N*=865	937<br>(297-2175)<br>*N*=421	1200<br>(654-1880)<br>*N*=1153	6204<br>(1356-11394)<br>*N*=261	1350<br>(346-3556)<br>*N*=1494	1892<br>(589-4057)<br>*N*=2299
dhps	581	5318<br>(1544-10153)<br>*N*=929	6376<br>(2500-16036)<br>*N*=446	2167<br>(1149-3282)<br>*N*=1161	13468<br>(2509-27526)<br>*N*=263	3732<br>(826-11618)<br>*N*=1743	6662<br>(2139-13428)<br>*N*=2473
dhps	613	3564<br>(966-7732)<br>*N*=912	5119<br>(1952-14158)<br>*N*=441	962<br>(568-1504)<br>*N*=1097	4769<br>(1088-15201)<br>*N*=258	2587<br>(563-8876)<br>*N*=1645	4536<br>(1338-9105)<br>*N*=2352

Gene	Amino acid(s)	Angola	Eswatini	Mozambique	Namibia	South Africa	Zambia
exo	415	873<br>(371-1874)<br>*N*=849	724<br>(230-1553)<br>*N*=405	1053<br>(634-1677)<br>*N*=1144	3478<br>(718-5748)<br>*N*=257	1103<br>(323-2754)<br>*N*=1472	1282<br>(409-3364)<br>*N*=2282
fd	193	870<br>(356-1932)<br>*N*=848	701<br>(224-1824)<br>*N*=401	1061<br>(580-1654)<br>*N*=1147	4314<br>(972-7211)<br>*N*=256	1280<br>(348-3138)<br>*N*=1302	1398<br>(454-2836)<br>*N*=2215
k13	210,250	8102<br>(2179-17000)<br>*N*=920	9605<br>(3939-26117)<br>*N*=444	1220<br>(729-1957)<br>*N*=1109	14276<br>(2890-28737)<br>*N*=259	5907<br>(1633-19148)<br>*N*=1734	8523<br>(3281-17382)<br>*N*=2405
k13	310,360	3028<br>(942-6320)<br>*N*=911	3498<br>(1363-10024)<br>*N*=444	986<br>(538-1614)<br>*N*=1106	3298<br>(828-8043)<br>*N*=257	2286<br>(590-7138)<br>*N*=1571	3603<br>(1285-6760)<br>*N*=2360
k13	436,437,441,442<br>446,449,452,454<br>458	1398<br>(501-3568)<br>*N*=874	1126<br>(344-2683)<br>*N*=431	1139<br>(630-1806)<br>*N*=1145	8770<br>(1788-15973)<br>*N*=259	1572<br>(406-4557)<br>*N*=1523	2146<br>(657-4652)<br>*N*=2314
k13	469,476,479,480<br>481,483,485,493<br>494	4046<br>(1156-7277)<br>*N*=928	3888<br>(1591-10981)<br>*N*=445	1753<br>(943-2637)<br>*N*=1156	13560<br>(2426-22203)<br>*N*=260	3314<br>(790-8774)<br>*N*=1736	4967<br>(1612-8802)<br>*N*=2470
k13	504,510,515	1900<br>(584-3824)<br>*N*=893	2384<br>(886-6292)<br>*N*=439	586<br>(351-900)<br>*N*=1080	2358<br>(582-6682)<br>*N*=248	1662<br>(442-4685)<br>*N*=1606	2240<br>(748-4357)<br>*N*=2292
k13	520,522,527	4832<br>(1324-8750)<br>*N*=930	4192<br>(1812-12557)<br>*N*=445	1392<br>(776-2047)<br>*N*=1154	18111<br>(3143-26790)<br>*N*=261	3866<br>(893-9906)<br>*N*=1739	5816<br>(1970-10135)<br>*N*=2469
k13	537,538,539,540<br>543,544,546,553	2004<br>(655-5064)<br>*N*=888	1515<br>(494-3775)<br>*N*=431	774<br>(453-1198)<br>*N*=1140	11887<br>(2425-21661)<br>*N*=260	2138<br>(477-6106)<br>*N*=1536	2999<br>(864-5890)<br>*N*=2333
k13	555,561,568,574<br>575,578,580,581<br>584	15567<br>(4386-36194)<br>*N*=931	17298<br>(6871-40664)<br>*N*=445	2005<br>(1121-3121)<br>*N*=1157	43306<br>(7266-85706)<br>*N*=263	10868<br>(2950-33301)<br>*N*=1787	16856<br>(6303-32593)<br>*N*=2481
k13	596,597,598,599<br>605,607,613	12772<br>(3183-31706)<br>*N*=922	15077<br>(5644-36082)<br>*N*=443	1248<br>(686-1952)<br>*N*=1110	26299<br>(5145-63172)<br>*N*=263	8830<br>(2498-27836)<br>*N*=1735	13018<br>(4956-27428)<br>*N*=2411
k13	622,631,641,656<br>659,661,662,667<br>673,675,690	767<br>(316-1719)<br>*N*=833	578<br>(194-1358)<br>*N*=400	914<br>(463-1471)<br>*N*=1140	4357<br>(903-7642)<br>*N*=258	881<br>(237-2207)<br>*N*=1348	1223<br>(406-2348)<br>*N*=2168
mdr1	86	439<br>(226-915)<br>*N*=746	376<br>(143-814)<br>*N*=352	723<br>(406-1136)<br>*N*=1122	1901<br>(478-3916)<br>*N*=255	623<br>(210-1440)<br>*N*=1220	716<br>(250-1514)<br>*N*=2023
mdr1	182,184,186	3238<br>(1030-8468)<br>*N*=896	2544<br>(854-6443)<br>*N*=440	1406<br>(743-2316)<br>*N*=1154	19979<br>(3396-32651)<br>*N*=259	3204<br>(636-10466)<br>*N*=1476	4756<br>(1385-9925)<br>*N*=2355
mdr1	371	1213<br>(475-2922)<br>*N*=879	910<br>(343-2250)<br>*N*=436	1632<br>(896-2600)<br>*N*=1154	6634<br>(1388-10499)<br>*N*=259	1347<br>(334-3839)<br>*N*=1449	2058<br>(649-4487)<br>*N*=2316
mdr1	1034,1042	2307<br>(792-6228)<br>*N*=903	1896<br>(671-4719)<br>*N*=442	2079<br>(1135-3386)<br>*N*=1154	14256<br>(2800-25016)<br>*N*=259	2399<br>(581-7708)<br>*N*=1610	3806<br>(1148-8991)<br>*N*=2388
mdr1	1246	1992<br>(718-5122)<br>*N*=892	1705<br>(526-4040)<br>*N*=438	1971<br>(1063-3138)<br>*N*=1155	11414<br>(2890-24786)<br>*N*=260	2122<br>(522-6962)<br>*N*=1592	3272<br>(972-8076)<br>*N*=2369

**Gene**	**Amino acid(s)**	**Angola**	**Eswatini**	**Mozambique**	**Namibia**	**South Africa**	**Zambia**
mdr2	463,484,492,515	1056<br>(400-2599)<br>*N*=843	897<br>(267-2348)<br>*N*=393	1300<br>(719-2060)<br>*N*=1147	5855<br>(1596-13360)<br>*N*=259	1357<br>(353-3970)<br>*N*=1385	1652<br>(468-3645)<br>*N*=2204
pib7	1484	1304<br>(493-2776)<br>*N*=881	1071<br>(280-2459)<br>*N*=429	1124<br>(626-1848)<br>*N*=1150	5231<br>(1106-8449)<br>*N*=259	1503<br>(393-3810)<br>*N*=1540	1913<br>(628-4137)<br>*N*=2327

Supplemental Table 2. Proportion of samples with non-synonymous *kelch13* mutations by mutation and participating Elimination 8 countries. Mutations were categorized using the current World Health Organization classification framework as validated, candidate, or unvalidated markers of artemisinin partial resistance (ART-R).[40] Non-zero values are shown in bold. Proportions are calculated as the number of samples with a genotype (pure or mixed) divided by the total number of genotyped infections.

	Angola	Eswatini	Mozambique	Namibia	South Africa	Zambia
**Validated ART-R markers**						
**P553L**	0% (*N*=754)	**0.97% (*N*=412)**	0% (*N*=840)	0% (*N*=233)	**0.07% (*N*=1,522)**	0% (*N*=2,254)
**P574L**	**0.13% (*N*=781)**	0% (*N*=424)	0% (*N*=854)	**1.27% (*N*=236)**	**0.23% (*N*=1,770)**	**0.67% (*N*=2,400)**
**A675V**	0% (*N*=711)	0% (*N*=382)	0% (*N*=847)	**1.3% (*N*=231)**	0% (*N*=1,334)	**0.1% (*N*=2,091)**
**Candidate ART-R markers**						
**R515K**	0% (*N*=762)	0% (*N*=418)	0% (*N*=798)	**0.9% (*N*=221)**	0% (*N*=1,589)	0% (*N*=2,214)
**P441L**	**0.4% (*N*=742)**	0% (*N*=412)	0% (*N*=846)	**32.9% (*N*=231)**	0% (*N*=1,509)	**3% (*N*=2,235)**
**Mutations lacking evidence of ART-R**						
**K479I**	0% (*N*=781)	0% (*N*=424)	0% (*N*=853)	**0.86% (*N*=233)**	0% (*N*=1,719)	**0.04% (*N*=2,389)**
**S485N**	0% (*N*=781)	0% (*N*=424)	0% (*N*=853)	0% (*N*=233)	0% (*N*=1,719)	**0.21% (*N*=2,389)**
**V494I**	0% (*N*=781)	0% (*N*=424)	**0.12% (*N*=853)**	0% (*N*=233)	**1.11% (*N*=1,719)**	0% (*N*=2,389)
**N537S**	0% (*N*=754)	0% (*N*=412)	0% (*N*=840)	0% (*N*=233)	**0.39% (*N*=1,522)**	**0.04% (*N*=2,254)**
**R539I**	0% (*N*=754)	0% (*N*=412)	0% (*N*=840)	0% (*N*=233)	0% (*N*=1,522)	**0.22% (*N*=2,254)**
**A578S**	**0.77% (*N*=781)**	0% (*N*=424)	**0.7% (*N*=854)**	**0.42% (*N*=236)**	**2.37% (*N*=1,770)**	**0.92% (*N*=2,400)**
**Q613E**	**1.28% (*N*=781)**	0% (*N*=422)	**0.12% (*N*=823)**	0% (*N*=236)	0% (*N*=1,718)	**0.17% (*N*=2,331)**
**R622T**	0% (*N*=711)	0% (*N*=382)	0% (*N*=847)	**4.33% (*N*=231)**	0% (*N*=1,334)	**1.48% (*N*=2,091)**
**P667A**	**0.28% (*N*=711)**	0% (*N*=382)	0% (*N*=847)	**0.87% (*N*=231)**	0% (*N*=1,334)	**3.4% (*N*=2,091)**
**P667S**	0% (*N*=711)	0% (*N*=382)	0% (*N*=847)	**0.43% (*N*=231)**	0% (*N*=1,334)	**0.53% (*N*=2,091)**

Supplemental Table 3. Proportions of samples from each of the participating Elimination Eight countries with mutations in the dihydropteroate synthase (*dhps*) and dihydrofolate reductase (*dhfr*) genes. Proportions are calculated as the number of samples with a genotype (pure or mixed) divided by the total number of genotyped infections. Undetermined haplotypes, and haplotypes present in <0.5% of the samples in any country are excluded. Nonsynonymous mutations are underlined and in bold

	Angola	Eswatini	Mozambique	Namibia	South Africa	Zambia
dhfr **(51, 59, 108 ,164)**	*N*=748	*N*=421	*N*=840	*N*=232	*N*=1593	*N*=2282
N**RN**I	**2.94%**	**3.56%**	**3.93%**	**5.17%**	**1.32%**	**3.77%**
**I**C**N**I	**36.9%**	0%	**3.1%**	**8.19%**	**1%**	**7.1%**
**IRN**I	**87.17%**	**99.05%**	**98.33%**	**96.12%**	**99.56%**	**96.93%**
dhps **(431, 436, 437, 540, 581,613)**	*N*=622	*N*=400	*N*=668	*N*=224	*N*=1391	*N*=1869
ISAKAA	**3.38%**	**2.5%**	**4.79%**	0%	**1.65%**	**2.62%**
I**A**AKAA	**0.48%**	0%	**1.05%**	0%	**0.14%**	**0.27%**
IS**G**KAA	**69.61%**	2%	**0.75%**	**25.45%**	**2.95%**	**17.17%**
I**AG**KAA	**21.38%**	0%	0%	**2.23%**	0%	**0.91%**
I**AGE**AA	**2.09%**	0%	**0.15%**	0%	0%	**0.05%**
ISA**E**AA	0%	**0.75%**	**0.75%**	0%	**0.86%**	**0.7%**
IS**GE**AA	**32.48%**	**94.25%**	**93.41%**	**74.11%**	**97.63%**	**89.73%**
IS**GEG**A	**1.29%**	**6%**	**0.75%**	**11.16%**	**0.36%**	**3.42%**
**V**A**G**K**GS**	0%	**0.75%**	0%	0%	0%	0%
dhps dhfr	*N*=525	*N*=400	*N*=659	*N*=218	*N*=1382	*N*=1827
ISAKAA **I**C**N**I	**0.76%**	0%	0%	0%	**0.07%**	**0.22%**
ISAKAA **IRN**I	**1.9%**	**2.5%**	**4.55%**	0%	**1.59%**	**2.35%**
I**A**AKAA **IRN**I	**0.38%**	0%	**0.91%**	0%	**0.14%**	**0.22%**
IS**G**KAA N**RN**I	**1.14%**	0%	0%	**0.92%**	0%	**0.05%**
IS**G**KAA **I**C**N**I	**16.38%**	0%	0%	**4.59%**	0%	**0.33%**
IS**G**KAA **IRN**I	**53.52%**	**2%**	**0.61%**	**21.56%**	**2.68%**	**15.27%**
I**AG**KAA **I**C**N**I	**3.81%**	0%	0%	0%	0%	**0.05%**
I**AG**KAA **IRN**I	**14.29%**	0%	0%	**2.29%**	0%	**0.93%**
I**AGE**AA **IRN**I	**1.52%**	0%	0%	0%	0%	**0.05%**
ISA**E**AA **IRN**I	0%	**0.75%**	**0.61%**	0%	**0.87%**	**0.6%**
IS**GE**AA N**RN**I	**0.38%**	**3.5%**	**2.88%**	**1.83%**	**0.72%**	**2.63%**
IS**GE**AA **I**C**N**I	**3.43%**	0%	**2.28%**	**2.29%**	**0.65%**	**4.27%**
IS**GE**AA **IRN**I	**25.9%**	**93.25%**	**91.81%**	**72.02%**	**97.32%**	**86.21%**
IS**GEG**A **IRN**I	**1.71%**	**6%**	**0.61%**	**11.01%**	**0.29%**	**3.39%**
**V**A**G**K**GS** **IRN**I	0%	**0.75%**	0%	0%	0%	0%

Supplemental Table 4. Proportions of samples from each of the participating Elimination Eight countries with mutations in the chloroquine resistance transporter (*crt*) gene.

Proportions are calculated as the number of samples with a genotype (pure or mixed) divided by the total number of genotyped infections. Only mutations/haplotypes present in >0.5% of the samples in any country are shown

	**Angola**	**Eswatini**	**Mozambique**	**Namibia**	**South Africa**	**Zambia**
72-76 CVIET	18.82% (*N*=781)	0.47% (*N*=425)	0.12% (*N*=862)	0.85% (*N*=235)	0.06% (*N*=1,739)	0.21% (*N*=2,405)
A220S	12.56% (*N*=223)	5.26% (*N*=95)	0.14% (*N*=732)	0% (*N*=130)	0% (*N*=545)	0.13% (*N*=747)
I356T	13.69% (*N*=745)	0% (*N*=423)	0% (*N*=854)	0% (*N*=228)	0% (*N*=1,564)	0% (*N*=2,300)

Supplemental Table 5. Proportions of samples from each of the participating Elimination Eight countries with mutations in the multidrug resistance 1 (*mdr1*) gene

Proportions are calculated as the number of samples with a genotype (pure or mixed) divided by the total number of genotyped infections.

	Angola	Eswatini	Mozambique	Namibia	South Africa	Zambia
**86**	*N*=628	*N*=336	*N*=836	*N*=228	*N*=1,207	*N*=1,949
N	99.2%	100%	99.88%	100%	99.92%	100%
Y	2.07%	0%	0.12%	0%	0.99%	0.21%
**184**	*N*=760	*N*=421	*N*=854	*N*=232	*N*=1,460	*N*=2,273
F	52.11%	66.27%	72.13%	51.72%	67.47%	52.27%
**1246**	*N*=628	*N*=336	*N*=836	*N*=228	*N*=1,207	*N*=1,949
Y	2.07%	0%	0.12%	0%	0.99%	0.21%
**86, 184, 1246 haplotype**	*N*=621	*N*=335	*N*=827	*N*=224	*N*=1,164	*N*=1,937
NFD	51.05%	65.97%	71.95%	50.89%	67.7%	52.35%
NYD	79.07%	58.51%	70.25%	70.98%	69.85%	81.52%
YYD	1.13%	0%	0%	0%	0.09%	0%
