# Supplementary material for: Geographical Variation in Antimalarial Drug Resistance Marker Prevalence across the Southern African Elimination Eight Region
Source: Am J Trop Med Hyg. 2026 Oct 6:tpmd260299. Online ahead of print. doi: 10.4269/ajtmh.26-0299 (PMC13675676; doi:10.4269/ajtmh.26-0299)
Supplement: Supplementary file 1 [file tpmd260299.SD1.pdf]

Supplement for:

Geographical Variation in Antimalarial Drug Resistance Marker Prevalence Across the Southern African Elimination 8 Region

Authors:

Jaishree Raman*[1,2,3], Andrés Aranda-Díaz*[4,5,6#], Maxwell Mabona[1], Mukosha Chisenga[7], Maria Florinda João[8], Domingos Jandondo[9], Pedro Rafael Dimbu[8], Nomcebo Nhlengethwa[10], Sabelo V. Dlamini[11], Lydia Eloff[12,13], Stark Katokele[14], Davis R. Mumbengegwi[13], Qedusizi Nyawo[15], Mbavhalelo Shandukani[16], Sydney Mwanza[17], Moonga Hawela[18], Simone Boene[6,19,20], Arlindo Chidimatembue[19], Bernardete Rafael[21], Eduard Rovira-Vallbona[6], Brighton Mangena[7], Sonja B. Lauterbach[1], Takalani I. Makhanthisa[1], Hazel Gwarinda[1], Blaženka D Letinić[1], Faith De Amaral[4], Isobel Routledge[5], Beatriz Arregui-Gallego[6], Mishalan Moodley[22], Jonathan Featherstone[22], Power B. Tshikae[15], Arshad Ismail[22], Jose Franco Martins[8], Quinton Dlamini[10], Baltazar Candrinho[21], Petrina Uusiku[14], Ednah Baloyi[16], Busiku Hamainza[18], Alfredo Mayor[6,19,20,23,24], Bryan Greenhouse[4], Amy Wesolowski[25], Chadwick Sikaala[7], John Chimumbwa[$7], Jennifer L. Smith[$5,26]

Affiliations:

[1] Laboratory for Antimalarial Resistance Monitoring and Malaria Operational Research, Centre of Emerging Zoonotic and Parasitic Diseases, National Institute for Communicable Diseases, Johannesburg, South Africa

[2] Wits Research Institute for Malaria, University of Witwatersrand, Johannesburg, South Africa

[3] University of Pretoria Institute for Sustainable Malaria Control, University of Pretoria, Pretoria, South Africa

[4] EPPIcenter Research Program, Department of Medicine, University of California, San Francisco, United States of America

[5] Malaria Elimination Initiative, Global Health Group, University of California, San Francisco, United States of America

[6] ISGlobal, Barcelona, Spain

[7] SADC Malaria Elimination Eight Secretariat, Windhoek, Namibia

[8] Programa Nacional do Controlo da Malária, Luanda, Angola

[9] Instituto Nacional de Investigação em Saúde, Luanda, Angola

[10]National Malaria Programme, Manzini, Eswatini
[11] Department of Environmental Health Sciences, University of Eswatini, Mbabane, Eswatini
[12] Department of Biochemistry, Microbiology, and Biotechnology, School of Science, University of Namibia, Windhoek, Namibia
[13] Malaria Operational Research Program, Multidisciplinary Research Services, University of Namibia, Windhoek, Namibia
[14] National Vector-Borne Disease Control Program, Ministry of Health and Social Services, Windhoek, Namibia
[15] KwaZulu-Natal Provincial Malaria Control Program, Jozini, KwaZulu-Natal, South Africa
[16] National Department of Health, Pretoria, South Africa
[17] National Health Research and Training Institute, Ndola, Zambia
[18] National Malaria Elimination Centre, Ministry of Health, Lusaka, Zambia
[19] Centro de Investigação em Saúde de Manhiça, Manhiça, Mozambique
[20] Facultat de Medicina i Ciències de la Salut, Universitat de Barcelona, Barcelona, Spain
[21] Programa Nacional do Controlo da Malária, Maputo, Mozambique
[22] Sequencing Core Facility, National Institute for Communicable Diseases, Johannesburg, South Africa
[23] Faculdade de Medicina, Universidade Eduardo Mondlane, Maputo, Mozambique
[24] Spanish Consortium for Research in Epidemiology and Public Health (CIBERESP), Madrid, Spain
[25] Department of Epidemiology Johns Hopkins University Bloomberg School of Public Health, Baltimore, Maryland, United States of America
[26] Department of Epidemiology & Biostatistics, University of California, San Francisco, United States of America

*: contributed equally
[$]: co-senior authors
[#] corresponding author: Andrés Aranda-Díaz
E-mail: andres.aranda@isglobal.org
Address: Carrer del Rosselló, 132, 5-2, 08036 Barcelona, Spain

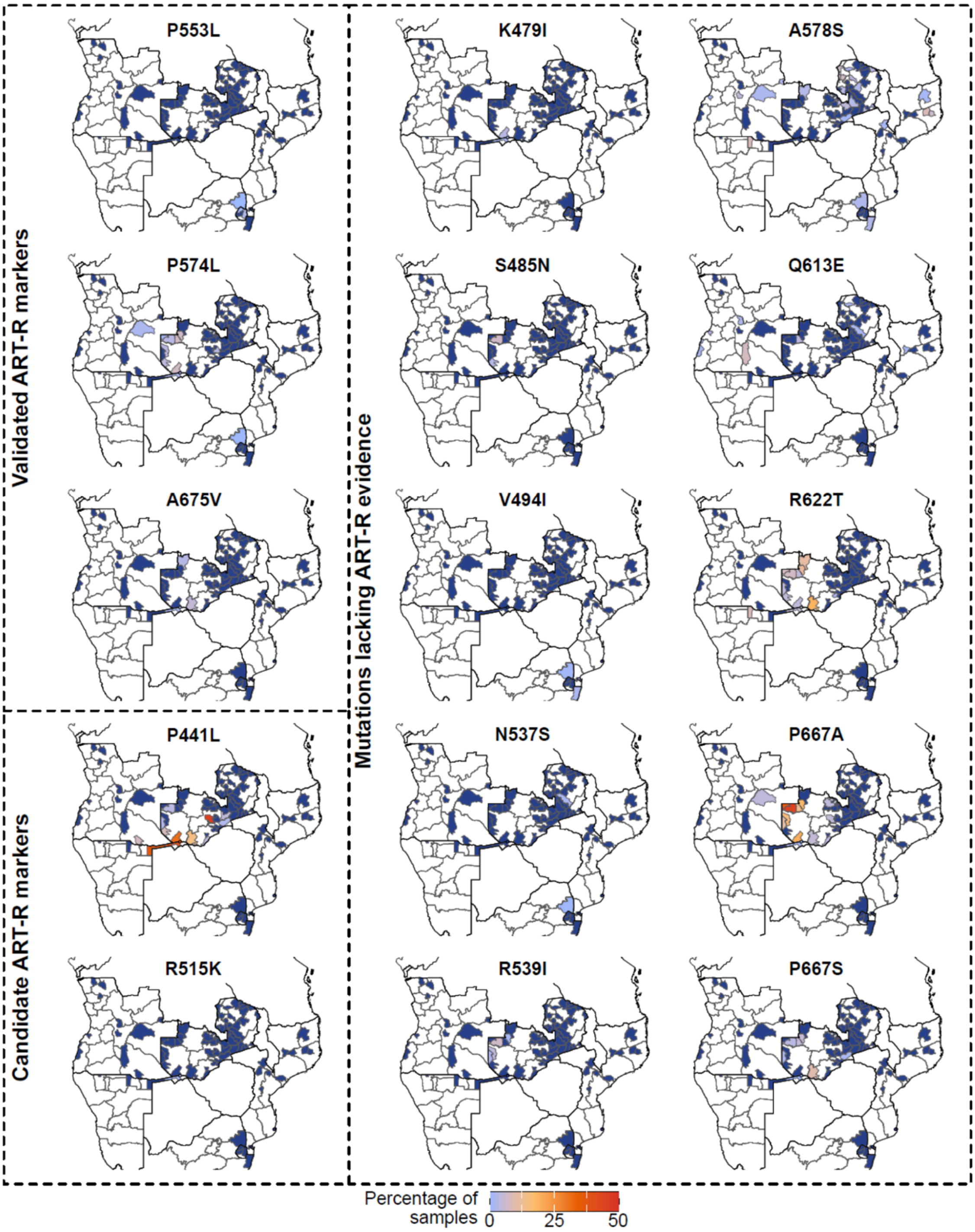

Figure S1. Proportion of samples with non-synonymous *kelch13* mutations in the study districts from the six participating Elimination Eight countries. Proportions are

calculated as the number of samples with a genotype (pure or mixed) divided by the total number of genotyped infections. Dark blue shading indicates absence of a marker.

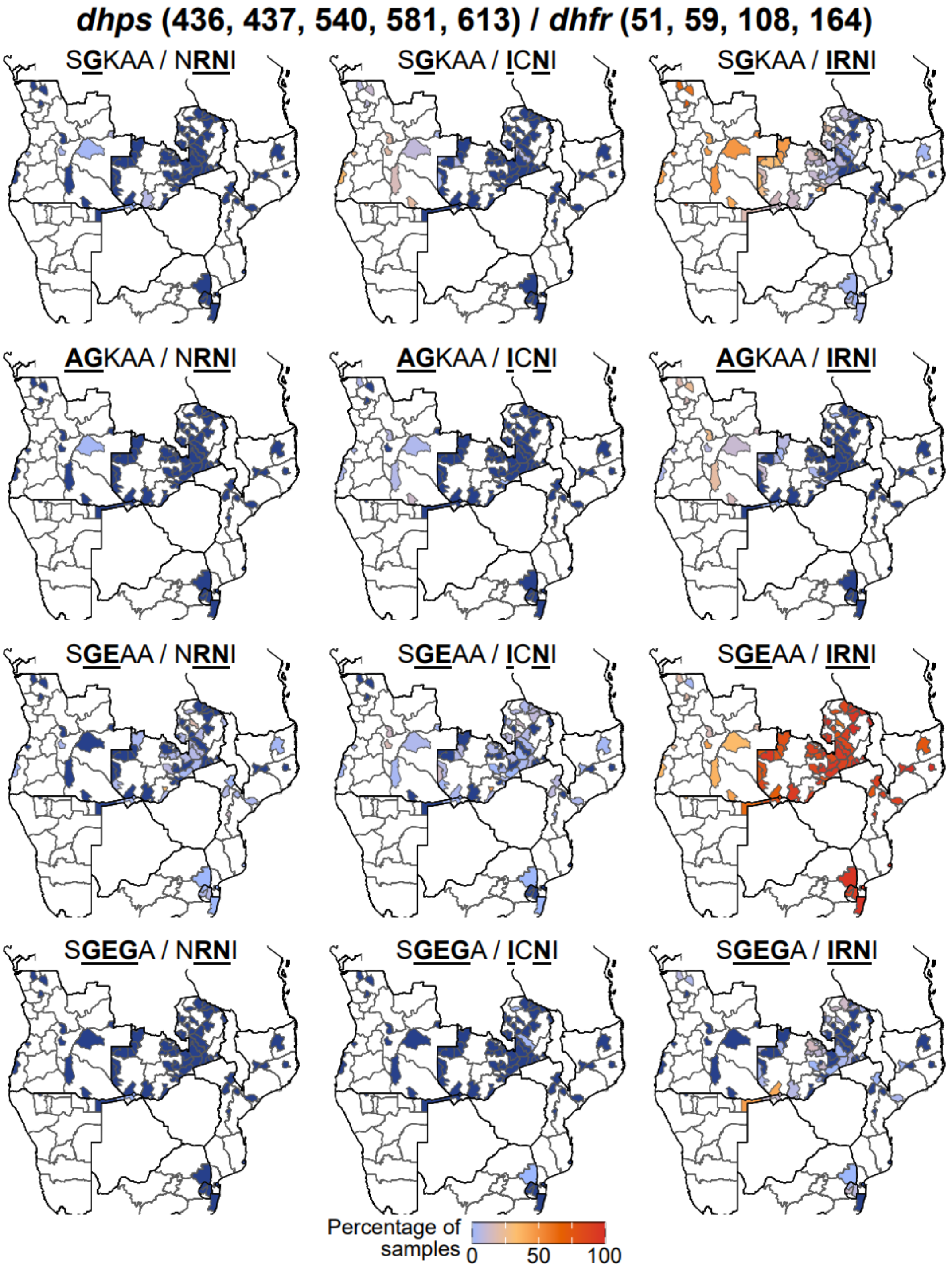

Figure S2. Proportion of samples from the study districts in the six participating Elimination Eight countries carrying different *dhps* and *dhfr* combined haplotypes. Proportions are calculated as the number of samples with a haplotype (pure or mixed)

divided by the total number of genotyped infections. Dark blue shading indicates absence of a marker.

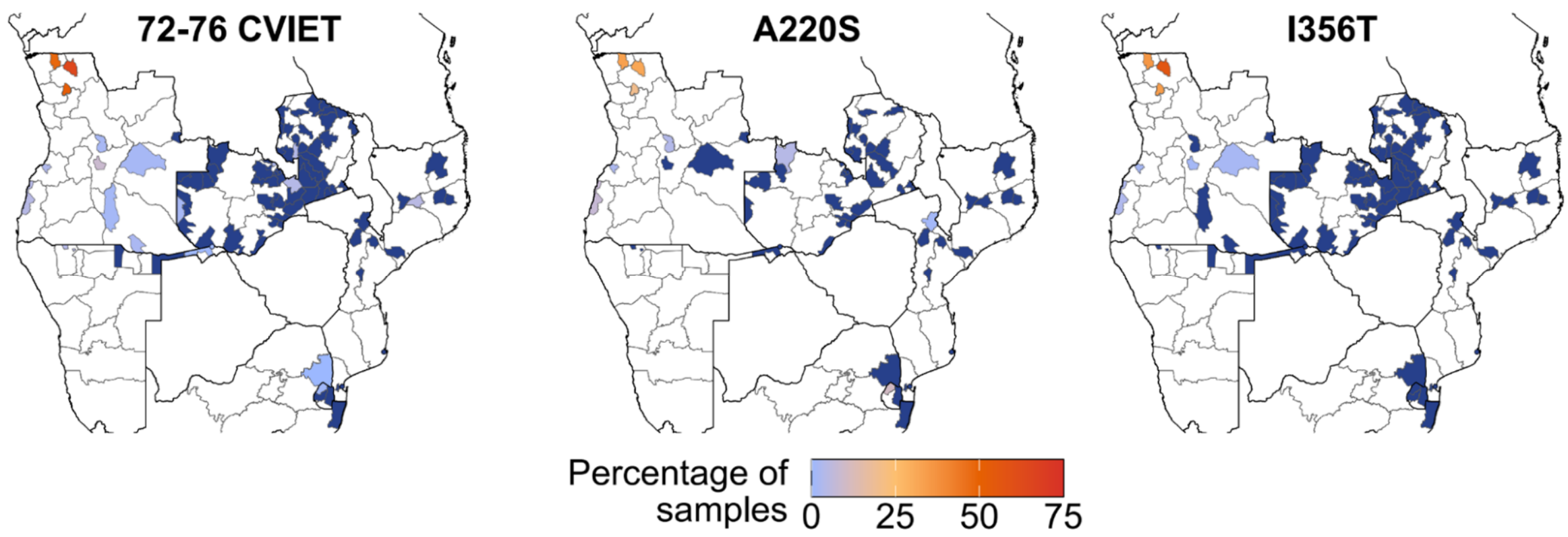

Figure S3. Proportion of samples from the study districts in the six participating Elimination Eight countries with mutations in the *crt* gene. Dark blue shading indicates absence of a marker.

Table S1. depth at reported SNP loci across participating countries. Depth of coverage is reported per amplicon, with SNPs sharing a single amplicon summarized together. Values shown are median read depth (interquartile range) and the number of samples with non-zero coverage at each locus.

Gene	Codon(s)	Angola	Eswatini	Mozambique	Namibia	South Africa	Zambia
**PF3D7_1322700**	**236**	731 (246-1845) *N*=754	677 (210-1814), *N*=358	1149 (539-1968), *N*=1074	3985 (1294-10022), *N*=239	999 (270-2918), *N*=1166	1268 (380-3287), *N*=2030
**arps10**	**127,128**	1898 (721-4375), *N*=905	1554 (492-3463), *N*=442	1336 (723-2228), *N*=1155	8286 (1574-13227), *N*=260	2004 (506-5402), *N*=1455	3117 (1045-5898), *N*=2365
**coronin**	**50**	1801 (602-4391), *N*=883	1343 (446-3526), *N*=435	1601 (948-2506), *N*=1152	10756 (2123-19698), *N*=259	2504 (556-7129), *N*=1387	3038 (854-7528), *N*=2304
**coronin**	**100, 107**	14603 (4108-31305), *N*=931	17542 (6902-40333), *N*=446	2857 (1645-4295), *N*=1162	38154 (6293-76162), *N*=261	8678 (1872-30077), *N*=1780	17960 (5996-34286), *N*=2479
**crt**	**48,52, 72-76**	6590 (1736-13080), *N*=929	6373 (2696-17831), *N*=446	2340 (1248-3688), *N*=1162	26507 (3570-36094), *N*=262	4863 (1050-15001), *N*=1705	8099 (2691-15814), *N*=2480
**crt**	**93**	6648 (1804-13084), *N*=929	6373 (2696-17831), *N*=446	2338 (1245-3680), *N*=1162	26507 (3570-36094), *N*=262	4929 (1053-15099), *N*=1705	8084 (2687-15818), *N*=2480
**crt**	**97,101**	2285 (588-5106), *N*=897	3133 (1044-8500), *N*=439	685 (383-1140), *N*=1080	3115 (680-8802), *N*=250	2094 (502-6010), *N*=1550	2755 (863-5786), *N*=2296
**crt**	**145**	586 (221-1411), *N*=741	566 (200-1646), *N*=381	551 (268-966), *N*=1071	1978 (668-5960), *N*=239	666 (214-2059), *N*=1309	784 (257-2148), *N*=2021
**crt**	**218,220**	112 (74-204), *N*=262	123 (76-251), *N*=99	215 (130-388), *N*=895	186 (93-539), *N*=146	165 (93-352), *N*=549	143 (80-269), *N*=773
**crt**	**343,350, 353,356**	944 (298-2048), *N*=826	1349 (456-4258), *N*=431	356 (212-569), *N*=1030	1014 (283-3642), *N*=238	919 (252-2669), *N*=1431	1180 (422-2665), *N*=2177
**dhfr**	**16,51, 59**	1120 (441-2692), *N*=873	965 (282-1968), *N*=423	1268 (723-1962), *N*=1152	5696 (1122-8709), *N*=257	1218 (314-3235), *N*=1506	1694 (545-3512), *N*=2304
**dhfr**	**108,164**	3620 (1268-8393), *N*=913	2891 (967-6588), *N*=441	1170 (618-1944), *N*=1155	16357 (2778-25088), *N*=261	3494 (832-10352), *N*=1636	5296 (1728-9559), *N*=2396
**dhfr**	**185**	1099 (412-2081), *N*=889	1246 (525-4321), *N*=436	414 (253-662), *N*=1066	1016 (240-2141), *N*=246	1020 (304-2904), *N*=1559	1575 (597-3492), *N*=2321
**dhps**	**431,436, 437**	4005 (1396-9850), *N*=917	3305 (1274-7945), *N*=446	1577 (892-2581), *N*=1152	11996 (3088-29024), *N*=262	3549 (903-10788), *N*=1661	6605 (2251-13023), *N*=2419
**dhps**	**540**	1086 (444-2577), *N*=865	937 (297-2175), *N*=421	1200 (654-1880), *N*=1153	6204 (1356-11394), *N*=261	1350 (346-3556), *N*=1494	1892 (589-4057), *N*=2299
**dhps**	**581**	5318 (1544-10153), *N*=929	6376 (2500-16036), *N*=446	2167 (1149-3282), *N*=1161	13468 (2509-27526), *N*=263	3732 (826-11618), *N*=1743	6662 (2139-13428), *N*=2473
**dhps**	**613**	3564	5119	962	4769	2587	4536

		(966-7732), *N*=912	(1952-14158), *N*=441	(568-1504), *N*=1097	(1088-15201), *N*=258	(563-8876), *N*=1645	(1338-9105), *N*=2352
**exo**	**415**	873 (371-1874), *N*=849	724 (230-1553), *N*=405	1053 (634-1677), *N*=1144	3478 (718-5748), *N*=257	1103 (323-2754), *N*=1472	1282 (409-3364), *N*=2282
**fd**	**193**	870 (356-1932), *N*=848	701 (224-1824), *N*=401	1061 (580-1654), *N*=1147	4314 (972-7211), *N*=256	1280 (348-3138), *N*=1302	1398 (454-2836), *N*=2215
**k13**	**210,250**	8102 (2179-17000), *N*=920	9605 (3939-26117), *N*=444	1220 (729-1957), *N*=1109	14276 (2890-28737), *N*=259	5907 (1633-19148), *N*=1734	8523 (3281-17382), *N*=2405
**k13**	**310,360**	3028 (942-6320), *N*=911	3498 (1363-10024), *N*=444	986 (538-1614), *N*=1106	3298 (828-8043), *N*=257	2286 (590-7138), *N*=1571	3603 (1285-6760), *N*=2360
**k13**	**436,437,441, 442,446,449, 452,454,458**	1398 (501-3568), *N*=874	1126 (344-2683), *N*=431	1139 (630-1806), *N*=1145	8770 (1788-15973), *N*=259	1572 (406-4557), *N*=1523	2146 (657-4652), *N*=2314
**k13**	**469,476,479, 480,481,483, 485,493,494**	4046 (1156-7277), *N*=928	3888 (1591-10981), *N*=445	1753 (943-2637), *N*=1156	13560 (2426-22203), *N*=260	3314 (790-8774), *N*=1736	4967 (1612-8802), *N*=2470
**k13**	**504,510,515**	1900 (584-3824), *N*=893	2384 (886-6292), *N*=439	586 (351-900), *N*=1080	2358 (582-6682), *N*=248	1662 (442-4685), *N*=1606	2240 (748-4357), *N*=2292
**k13**	**520,522,527**	4832 (1324-8750), *N*=930	4192 (1812-12557), *N*=445	1392 (776-2047), *N*=1154	18111 (3143-26790), *N*=261	3866 (893-9906), *N*=1739	5816 (1970-10135), *N*=2469
**k13**	**537,538,539, 540,543,544, 546,553**	2004 (655-5064), *N*=888	1515 (494-3775), *N*=431	774 (453-1198), *N*=1140	11887 (2425-21661), *N*=260	2138 (477-6106), *N*=1536	2999 (864-5890), *N*=2333
**k13**	**555,561,568, 574,575,580, 581,584**	15567 (4386-36194), *N*=931	17298 (6871-40664), *N*=445	2005 (1121-3121), *N*=1157	43306 (7266-85706), *N*=263	10868 (2950-33301), *N*=1787	16856 (6303-32593), *N*=2481
**k13**	**596,597,598, 599,605,607, 613**	12772 (3183-31706), *N*=922	15077 (5644-36082), *N*=443	1248 (686-1952), *N*=1110	26299 (5145-63172), *N*=263	8830 (2498-27836), *N*=1735	13018 (4956-27428), *N*=2411
**k13**	**622,631,641, 656,659,661, 662,667,673, 675**	767 (316-1719), *N*=833	578 (194-1358), *N*=400	914 (463-1471), *N*=1140	4357 (903-7642), *N*=258	881 (237-2207), *N*=1348	1223 (406-2348), *N*=2168
**mdr1**	**86**	439 (226-915), *N*=746	376 (143-814), *N*=352	723 (406-1136), *N*=1122	1901 (478-3916), *N*=255	623 (210-1440), *N*=1220	716 (250-1514), *N*=2023
**mdr1**	**182,184, 186**	3238 (1030-8468), *N*=896	2544 (854-6443), *N*=440	1406 (743-2316), *N*=1154	19979 (3396-32651), *N*=259	3204 (636-10466), *N*=1476	4756 (1385-9925), *N*=2355
**mdr1**	**371**	1213 (475-2922), *N*=879	910 (343-2250), *N*=436	1632 (896-2600), *N*=1154	6634 (1388-10499), *N*=259	1347 (334-3839), *N*=1449	2058 (649-4487), *N*=2316
**mdr1**	**1034,1042**	2307 (792-6228), *N*=903	1896 (671-4719), *N*=442	2079 (1135-3386), *N*=1154	14256 (2800-25016), *N*=259	2399 (581-7708), *N*=1610	3806 (1148-8991), *N*=2388
**mdr1**	**1246**	1992 (718-5122), *N*=892	1705 (526-4040), *N*=438	1971 (1063-3138), *N*=1155	11414 (2890-24786), *N*=260	2122 (522-6962), *N*=1592	3272 (972-8076), *N*=2369
**mdr2**	**463,484, 492,515**	1056 (400-2599), *N*=843	897 (267-2348), *N*=393	1300 (719-2060), *N*=1147	5855 (1596-13360), *N*=259	1357 (353-3970), *N*=1385	1652 (468-3645), *N*=2204
**pib7**	**1484**	1304 (493-2776), *N*=881	1071 (280-2459), *N*=429	1124 (626-1848), *N*=1150	5231 (1106-8449), *N*=259	1503 (393-3810), *N*=1540	1913 (628-4137), *N*=2327

Table S2. Proportion of samples with non-synonymous *kelch13* mutations by mutation and participating Elimination 8 countries. Mutations were categorized using the current World Health Organization classification framework as validated, candidate, or unvalidated markers of artemisinin partial resistance (ART-R).[29] Non-zero values are shown in bold. Proportions are calculated as the number of samples with a genotype (pure or mixed) divided by the total number of genotyped infections.

	Angola	Eswatini	Mozambique	Namibia	South Africa	Zambia
**Validated ART-R markers**						
**P553L**	0% (N=754)	**0.97% (N=412)**	0% (N=840)	0% (N=233)	**0.07% (N=1,522)**	0% (N=2,254)
**P574L**	**0.13% (N=781)**	0% (N=424)	0% (N=854)	**1.27% (N=236)**	**0.23% (N=1,770)**	**0.67% (N=2,400)**
**A675V**	0% (N=711)	0% (N=382)	0% (N=847)	**1.3% (N=231)**	0% (N=1,334)	**0.1% (N=2,091)**
**Candidate ART-R markers**						
**R515K**	0% (N=762)	0% (N=418)	0% (N=798)	**0.9% (N=221)**	0% (N=1,589)	0% (N=2,214)
**P441L**	**0.4% (N=742)**	0% (N=412)	0% (N=846)	**32.9% (N=231)**	0% (N=1,509)	**3% (N=2,235)**
**Mutations lacking evidence of ART-R**						
**K479I**	0% (N=781)	0% (N=424)	0% (N=853)	**0.86% (N=233)**	0% (0/1,719)	**0.04% (N=2,389)**
**S485N**	0% (N=781)	0% (N=424)	0% (N=853)	0% (N=233)	0% (N=1,719)	**0.21% (N=2,389)**
**V494I**	0% (N=781)	0% (N=424)	**0.12% (N=853)**	0% (N=233)	**1.11% (N=1,719)**	0% (N=2,389)
**N537S**	0% (N=754)	0% (N=412)	0% (N=840)	0% (N=233)	**0.39% (N=1,522)**	**0.04% (N=2,254)**
**R539I**	0% (N=754)	0% (N=412)	0% (N=840)	0% (N=233)	0% (N=1,522)	**0.22% (N=2,254)**
**A578S**	**0.77% (N=781)**	0% (N=424)	**0.7% (N=854)**	**0.42% (N=236)**	**2.37% (N=1,770)**	**0.92% (N=2,400)**
**Q613E**	**1.28% (N=781)**	0% (N=422)	**0.12% (N=823)**	0% (N=236)	0% (N=1,718)	**0.17% (N=2,331)**
**R622T**	0% (N=711)	0% (N=382)	0% (N=847)	**4.33% (N=231)**	0% (N=1,334)	**1.48% (N=2,091)**
**P667A**	**0.28% (N=711)**	0% (N=382)	0% (N=847)	**0.87% (N=231)**	0% (N=1,334)	**3.4% (N=2,091)**
**P667S**	0% (N=711)	0% (N=382)	0% (N=847)	**0.43% (N=231)**	0% (N=1,334)	**0.53% (N=2,091)**

Table S3. Proportions of samples from each of the participating Elimination Eight countries with mutations in the *dhps* and *dhfr* genes. Proportions are calculated as the number of samples with a genotype (pure or mixed) divided by the total number of genotyped infections. Undetermined haplotypes, and haplotypes present in <0.5% of the samples in any country are excluded. Nonsynonymous mutations are underlined and in bold

	**Angola**	**Eswatini**	**Mozambique**	**Namibia**	**South Africa**	**Zambia**
dhfr **(51, 59, 108 ,164)**	N=748	N=421	N=840	N=232	N=1593	N=2282
N**RN**I	**2.94%**	**3.56%**	**3.93%**	**5.17%**	**1.32%**	**3.77%**
**I**C**N**I	**36.9%**	0%	**3.1%**	**8.19%**	**1%**	**7.1%**
**IRN**I	**87.17%**	**99.05%**	**98.33%**	**96.12%**	**99.56%**	**96.93%**
dhps **(431, 436, 437, 540, 581,613)**	N=622	N=400	N=668	N=224	N=1391	N=1869
ISAKAA	**3.38%**	**2.5%**	**4.79%**	0%	**1.65%**	**2.62%**
I**A**AKAA	**0.48%**	0%	**1.05%**	0%	**0.14%**	**0.27%**
IS**G**KAA	**69.61%**	2%	**0.75%**	**25.45%**	**2.95%**	**17.17%**
I**AG**KAA	**21.38%**	0%	0%	**2.23%**	0%	**0.91%**
I**AGE**AA	**2.09%**	0%	**0.15%**	0%	0%	**0.05%**
ISA**E**AA	0%	**0.75%**	**0.75%**	0%	**0.86%**	**0.7%**
IS**GE**AA	**32.48%**	**94.25%**	**93.41%**	**74.11%**	**97.63%**	**89.73%**
IS**GEG**A	**1.29%**	**6%**	**0.75%**	**11.16%**	**0.36%**	**3.42%**
**V**A**G**K**GS**	0%	**0.75%**	0%	0%	0%	0%
dhps dhfr	N=525	N=400	N=659	N=218	N=1382	N=1827
ISAKAA **I**C**N**I	**0.76%**	0%	0%	0%	**0.07%**	**0.22%**
ISAKAA **IRN**I	**1.9%**	**2.5%**	**4.55%**	0%	**1.59%**	**2.35%**
I**A**AKAA **IRN**I	**0.38%**	0%	**0.91%**	0%	**0.14%**	**0.22%**
IS**G**KAA N**RN**I	**1.14%**	0%	0%	**0.92%**	0%	**0.05%**
IS**G**KAA **I**C**N**I	**16.38%**	0%	0%	**4.59%**	0%	**0.33%**
IS**G**KAA **IRN**I	**53.52%**	**2%**	**0.61%**	**21.56%**	**2.68%**	**15.27%**
I**AG**KAA **I**C**N**I	**3.81%**	0%	0%	0%	0%	**0.05%**
I**AG**KAA **IRN**I	**14.29%**	0%	0%	**2.29%**	0%	**0.93%**
I**AGE**AA **IRN**I	**1.52%**	0%	0%	0%	0%	**0.05%**
ISA**E**AA **IRN**I	0%	**0.75%**	**0.61%**	0%	**0.87%**	**0.6%**
IS**GE**AA N**RN**I	**0.38%**	**3.5%**	**2.88%**	**1.83%**	**0.72%**	**2.63%**
IS**GE**AA **I**C**N**I	**3.43%**	0%	**2.28%**	**2.29%**	**0.65%**	**4.27%**
IS**GE**AA **IRN**I	**25.9%**	**93.25%**	**91.81%**	**72.02%**	**97.32%**	**86.21%**
IS**GEG**A **IRN**I	**1.71%**	**6%**	**0.61%**	**11.01%**	**0.29%**	**3.39%**
**V**A**G**K**GS** **IRN**I	0%	**0.75%**	0%	0%	0%	0%

Table S4. Proportions of samples from each of the participating Elimination Eight countries with mutations in the *crt* gene.

Proportions are calculated as the number of samples with a genotype (pure or mixed) divided by the total number of genotyped infections. Only mutations/haplotypes present in >0.5% of the samples in any country are shown

	**Angola**	**Eswatini**	**Mozambique**	**Namibia**	**South Africa**	**Zambia**
72-76 CVIET	18.82% (N=781)	0.47% (N=425)	0.12% (N=862)	0.85% (N=235)	0.06% (N=1,739)	0.21% (N=2,405)
A220S	12.56% (N=223)	5.26% (N=95)	0.14% (N=732)	0% (N=130)	0% (N=545)	0.13% (N=747)
I356T	13.69% (N=745)	0% (N=423)	0% (N=854)	0% (N=228)	0% (N=1,564)	0% (N=2,300)

Table S5. Proportions of samples from each of the participating Elimination Eight countries with mutations in the *mdr1* gene

Proportions are calculated as the number of samples with a genotype (pure or mixed) divided by the total number of genotyped infections.

	Angola	Eswatini	Mozambique	Namibia	South Africa	Zambia
**86**	N=628	N=336	N=836	N=228	N=1,207	N=1,949
N	99.2%	100%	99.88%	100%	99.92%	100%
Y	2.07%	0%	0.12%	0%	0.99%	0.21%
**184**	N=760	N=421	N=854	N=232	N=1,460	N=2,273
F	52.11%	66.27%	72.13%	51.72%	67.47%	52.27%
**1246**	N=628	N=336	N=836	N=228	N=1,207	N=1,949
Y	2.07%	0%	0.12%	0%	0.99%	0.21%
**86, 184, 1246 haplotype**	N=621	N=335	N=827	N=224	N=1,164	N=1,937
NFD	51.05%	65.97%	71.95%	50.89%	67.7%	52.35%
NYD	79.07%	58.51%	70.25%	70.98%	69.85%	81.52%
YYD	1.13%	0%	0%	0%	0.09%	0%
